# Molecular evolution of gas cavity in [NiFeSe] hydrogenases resurrected *in silico*

**DOI:** 10.1038/srep19742

**Published:** 2016-01-28

**Authors:** Takashi Tamura, Naoki Tsunekawa, Michiko Nemoto, Kenji Inagaki, Toshiyuki Hirano, Fumitoshi Sato

**Affiliations:** 1 Graduate School of Environmental and Life Science, Okayama University, Okayama, 700-8530, Japan; 2 Institute of Industrial Science, the University of Tokyo, Komaba 153-8505, Japan; 3 PRESTO, Japan Science and Technology Agency, Japan

## Abstract

Oxygen tolerance of selenium-containing [NiFeSe] hydrogenases (Hases) is attributable to the high reducing power of the selenocysteine residue, which sustains the bimetallic Ni–Fe catalytic center in the large subunit. Genes encoding [NiFeSe] Hases are inherited by few sulphate-reducing δ-proteobacteria globally distributed under various anoxic conditions. Ancestral sequences of [NiFeSe] Hases were elucidated and their three-dimensional structures were recreated *in silico* using homology modelling and molecular dynamic simulation, which suggested that deep gas channels gradually developed in [NiFeSe] Hases under absolute anaerobic conditions, whereas the enzyme remained as a sealed edifice under environmental conditions of a higher oxygen exposure risk. The development of a gas cavity appears to be driven by non-synonymous mutations, which cause subtle conformational changes locally and distantly, even including highly conserved sequence regions.

Several microorganisms use molecular hydrogen (H_2_) as an energy vector between and within cells in a process underpinned by [FeFe] and [NiFe] hydrogenases (Hases), which reversibly oxidise H_2_ to protons and electrons at high rates^1^. The [NiFeSe] Hases constitute a subgroup of [NiFe] Hases with selenocysteine (Sec) substituting one of the four Cys residues that hold the bimetallic Ni–Fe catalytic center in the large subunit. Sec is structurally identical to Cys, except for a sulphur-to-selenium substitution. Sec is designated as the 21^st^ genetically encoded amino acid because it is co-translationally inserted into proteins at an in-frame opal codon^2^. [NiFeSe] Hases have emerged as attractive catalysts for biotechnological applications because of their good ability to produce or oxidise hydrogen, particularly with tolerance for moderate O_2_ concentrations.

Thus far, few [NiFeSe] Hases have been characterized from the sulphate-reducing δ-proteobacteria, including those from *Desulfovibrio salexigens*^3^, *D. vulgaris*^4^,^5^ and *Desulfomicrobium baculatum*^6^. Several more homologues of [NiFeSe] Hases can be retrieved by a homology search of the genome sequences of cognate sulphate-reducing bacteria (SRB), although their annotated amino acid sequences are truncated by an in-frame opal codon at the Sec residue position of established [NiFeSe] Hases. Reading through the in-frame opal codon as Sec requires the complete set of Sec-translation machinery, which is referred to as a selenosome^7^ (Fig. 1). In prokaryotes, the selenosome is composed of four genes: (1) *selA*, which encodes selenocysteine synthase, (2) *selB*, which encodes the elongation factor SelB, (3) *selC*, which encodes a Sec-specific tRNA^UGA^, and (4) *selD*, which encodes selenophosphate synthetase. In addition, the nucleotide sequence directly after the in-frame opal codon must contain a *cis*-acting element known as the Sec insertion sequence (SECIS). The SECIS element folds itself into a characteristic stem-loop structure, upon which the elongation factor SelB binds and allows an L-Sec-tRNA^UGA^ molecule to bind to the in-frame opal codon. L-Sec-tRNA^UGA^ is generated by selenocysteine synthase, which catalyses the conversion of L-Ser-charged tRNA^UGA^ to L-Sec-tRNA^UGA^ using monoselenophosphate, ^−^Se-HPO_3_^−^. The cryptanalysis of the in-frame UGA codon requires molecular recognition between these selenosome members, setting a co-evolutionary constraint along their molecular diversification.

In the microbial world, the dissimilatory sulphate reducers are unique in utilising inorganic sulphate as the terminal electron acceptor. The most widespread SRB in nature are mesophilic, gram-negative and non-spore-forming prokaryotes. SRB include genera that incompletely oxidise organic compounds to the level of acetate and some genera that completely oxidise organic substances to CO_2_. The range of electron acceptors utilised by these organisms has also been extended from inorganic sulphate to other substances, including iron (Fe), uranium, halogenated aromatic compounds and oxygen^8^. Furthermore, some SRB are now increasingly perceived as microaerophiles rather than obligate anaerobes, as originally recognized^9^. Members of SRB genera are equally common in marine and freshwater environments and occupy a wide range of habitats, including subsurface aquifer systems, deep subsurface and the human intestine.

While SRBs have adapted to various anoxic environments, their vital enzymes also adapted to their environments through molecular evolution. Typically, prosthetic metalloenzymes, such as [NiFe] Hases and [NiFeSe] Hases, are localized to extracellular compartments, such as the periplasmic space or outer membrane, and are highly sensitive to the oxygenation risk. [NiFe] Hases are metalloenzymes in which a tunnel for gas substrate transfer has been described on the basis of crystallography, kinetics and molecular dynamics (MD) simulation^10^. H_2_ and molecular oxygen approach the active site of Hases through channels that connect the exterior to the metal center, which is buried inside the large subunit of the heterodimeric protein. In the present study, homology modelling and MD simulation were employed to recreate the ternary structures of extant and ancestral [NiFeSe] Hases, providing an illustrated interpretation of the consequences of the molecular evolution of [NiFeSe] Hases, which have widely diverged along with versatile SRB hosts under various anoxic environments. Transformation of the predicted cavity morphology implies that evolution was essentially directed, if it were allowed, to open a gas cavity presumably to improve H_2_ uptake efficiency. The mechanism of mutation-driven cavity transformation is discussed on the basis of the involvement of non-synonymously substituted amino acid residues, which account for only 5% of the aligned residues, dominating the conformational state of the other 95% of highly conserved sequence regions.

## Results

### Identification of putative [NiFeSe] Hases

**Figure 2:**
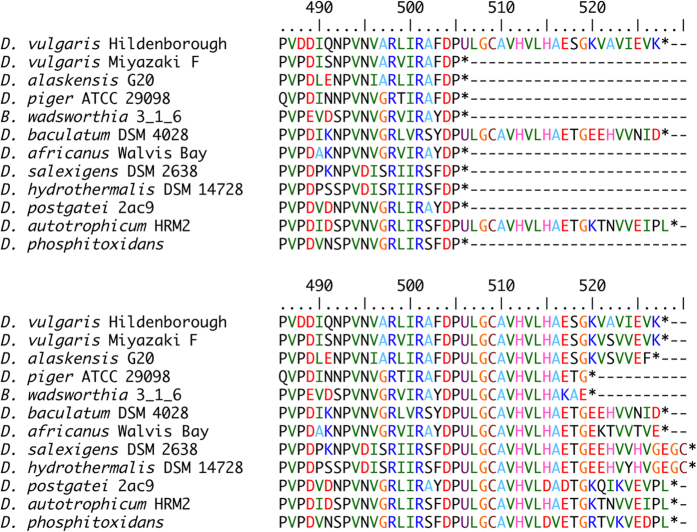
The C-terminal sequences of extant [NiFeSe] Hases, in which the in-frame opal codon was read through as Sec (U). Upper: Amino acid sequence annotated in GenBank/NCBI. Lower: Alternative translation of the in-frame opal codon as Sec (U).

**Figure 3:**
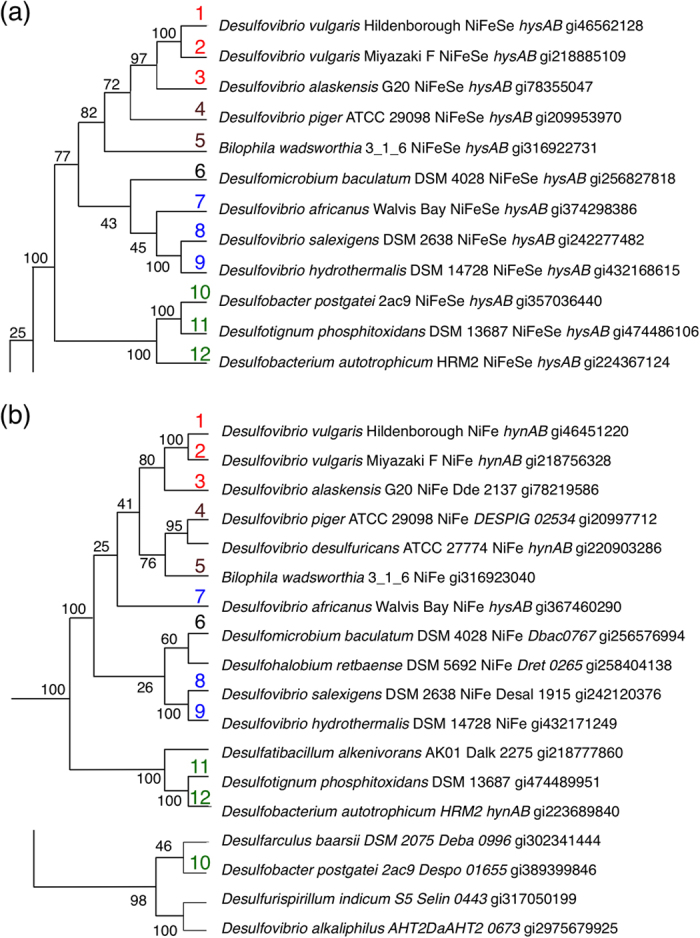
Phylogenetic relationships of (**a**) [NiFeSe] Hase and (**b**) [NiFe] Hase assemblages in a ML phylogenetic tree. Genes from sulphate-reducing bacteria of terrestrial origin (**1–3**), from clinical isolates (**4,5**), of marine vent origin (**7–9**), and of sediment origin (**10–12**) are numbered in red, brown, blue, and green, respectively. Scores designated at each branch represent the percent of the 300-times Bootstrap test.

[NiFe] and [NiFeSe] Hases contain Fe, nickel and Fe–sulphur clusters as redox-active metals. [NiFe] Hases have been isolated from various microorganisms, including strict aerobes, whereas [NiFeSe] Hases have been found only in a few *Desulfovibrio* strains. A homology search retrieved several [NiFeSe] Hase homologs with large subunits truncated by an in-frame opal codon corresponding to the Sec residue of established [NiFeSe] Hases. Read through of these opal codons as Sec revealed homologous sequences leading to second termination codons (Fig. 2). SECIS elements were also detected in these nucleotide sequences directly after the opal codon (Supplementary Fig. S1). To obtain insight into the molecular evolution of putative [NiFeSe] Hases, amino acid sequences of cognate [NiFe(Se)] Hases and their nucleotide sequences in microbial genomes were collected through pattern-specific phi-BLAST searches. Multiple alignments of 88 sequences of [NiFe] or [NiFeSe] Hase were performed using the MUSCLE^11^ and GUIDANCE with a PRANK algorithm for further refinement^12^. A maximum-likelihood (ML) phylogenetic tree was constructed on the basis of aligned sequences with 300 bootstrap values using MEGA 5.2.2 software^13^. The putative and established [NiFeSe] Hases were in a single clade with a bootstrap score of 100% at the branching point from other [NiFe] Hases (Fig. 3, Supplementary Fig. S2). This clade was reproduced even when the in-frame opal codon TGA was disguised as a cysteine-coding TGT codon, suggesting that the group was resolved from other [NiFe] Hases owing to the high homology shared by the entire sequence.

### Phylogenetic traits: Synchronicity and Coevolution

**Figure 4:**
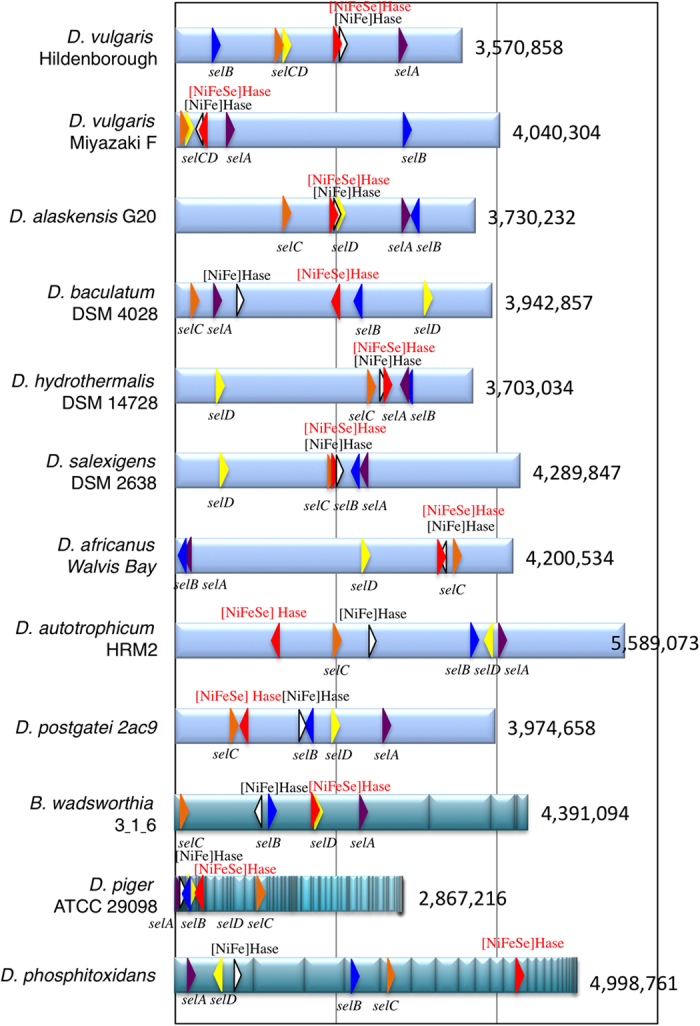
Physical genome maps. Location and coding directions are indicated by colored triangles representing *selA* (purple), *selB* (blue), *selC* (orange), *selD* (yellow), [NiFeSe] Hase (red) and [NiFe] Hase (white). Genomes of *B. wadsworthia 3_1_6, D. piger* and *D. phosphitoxidans* are draft sequences in which contigs are interrupted by gaps that remain to be elucidated for the completed genome sequencing.

Phylogenetic analysis of single assemblages of Hases may be poorly suitable for exploring the divergence of host bacterial phyla because prokaryotic phylogeny may be complicated by transformation, transduction and conjugation, collectively referred to as horizontal gene transfer. With Hases, horizontal gene transfer may be limited, if it ever occurs, to genetically close recipients because the enzymes require numerous *trans*-acting factors for maturation and localisation to the periplasmic space. Notably, [NiFe] and [NiFeSe] Hases underwent synchronized diversification, suggesting that the two paralogues also have parallel genetic traits (Fig. 3). One implication behind this synchronicity is that these Hases may share maturation and/or translocation machinery in their host bacteria. In case of [NiFeSe] Hases, decoding of the in-frame opal codon requires the selenosome genes *selA–D* and the SECIS element^7^. Because the biosynthetic machinery involves protein–protein and protein–RNA interactions to generate and insert L-Sec-t-RNA^UGA^ at the specific opal codon, the molecular diversification of these Hases is also under co-evolutionary constraint. In fact, significant similarities were observed in the evolutionary traits of [NiFeSe]-Hase-encoded *hysAB* and the selenosome members*, selA* and *selB* (Supplementary Fig. S3, S4). The tRNA^UGA^-encoding *selC* genes are so similar in their short sequences and predicted cloverleaf models that their individual phylogenetic traits were not clearly discernable except for three marine sediment-derived species (*D. autotrophicum* HRM2, *D. phosphitoxidans* and *D. postgatei* 2ac9), which presumably diverged at an earlier evolutionary stage (Supplementary Figs S5, S6). A striking contrast was observed in the diversification of *selD*, which appeared to be not in accord with that of *hynAB*, *hysAB*, *selA*, *selB* and *selC*, suggesting that the *selD* product, selenophosphate synthetase, did not coevolve with those genes (Supplementary Fig. S7). Because the catalytic function of the *selD* product is to provide ^−^Se-HPO_3_^−^, the metabolic precursor for L-Sec-tRNA biogenesis, direct contact with other selenosome members may not be essential. Significantly, physical maps revealed that genes encoding the Sec-translating machinery and the hydrogenase genes, *hysAB* ([NiFeSe] Hase) and *hynAB* ([NiFe] Hase), are scattered throughout the genome, assuring vertical inheritance of the host bacterial species (Fig. 4).

### Predicted history of cavity formation

**Figure 5:**
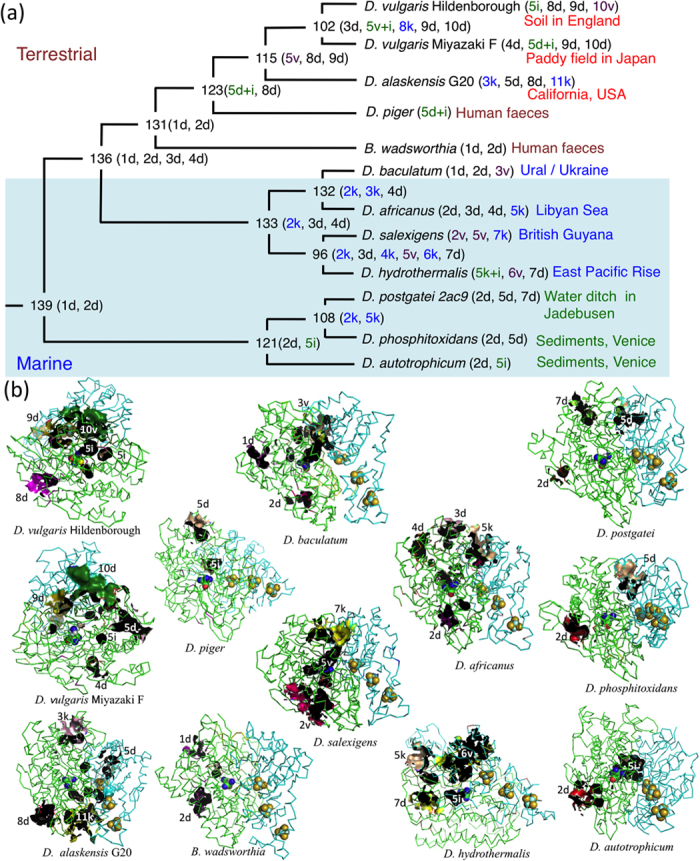
Molecular evolution of gas cavities in ancestral and extant [NiFeSe] Hases. (**a**) Diagram representation using cavity identity based on location and amino acid residues involved in the gas cavity and the designation of cavity size, dent (d) < crack (k) < crevice (v). The internal cavity (i) is an isolated cage near the Ni-Fe metal center. (**b**) Structures of modelled extant [NiFeSe] Hases including the MD models for *D. vulgaris* Hildenborough (2wpn.pdb)^34^ and *D. baculatum* (1cc1.pdb)^35^, which were also annealed on MD calculation. Gas cavities are designated as cavity identity and cavity size representation.

[NiFeSe] Hase is localized to the periplasmic space of SRB and the enzyme must have adapted to versatile anoxic environments. To elucidate the progressive development of the gas cavity of [NiFeSe] Hase from the common ancestor that first acquired Sec to the descendant homologues in presently living SRB, three-dimensional (3D) structures of putative and ancestral [NiFeSe] Hases were generated from the amino acid sequences by homology modelling and further optimisation by MD simulations (Supplementary Table S1). Gas channels and insulated internal cavities were identified by geometric computation. Cavities were identified based on the location in the MD models and amino acid residues involved; they were numbered in the order of appearance and classified as dent (d), crack (k), crevice (v), and internal cavity (i) (Table 1, Fig. 5). The sequence similarities designated as closer assemblages in the phylogenetic tree appear to be congruent to morphological similarities in the location and development of the cavities, profoundly implying that the primary sequence defines the ternary structure of the protein, as first suggested by Anfinsen^14^, and in this particular case, the void cavity generated on the protein surface (Supplementary Fig. S8). Our results indicated that the cavities of SRB isolated from deep sea and terrestrial environments as well as human faeces evolved in various ways, starting from the common ancestor 139, in which the bimetallic active center was sealed from the exterior. The surface dent and the internal cavity (5d + 5i) and their connected crevices (5v) in the intermediate ancestors 123 and 115, respectively, were generated besides the surface dents (1d, 2d) in the precedent common ancestors 136 and 131, and separated by the formation of internal cavities in [NiFeSe] Hases in *D. vulgaris* Hildenborough (5i) and *D. vulgaris* Miyazaki F (5d + 5i) strains.

*D. baculatum*, *D. africanus*, *D. salexigens* and *D. hydrothermalis* inhabit anaerobic aquatic environments and their [NiFeSe] Hases share a high sequence homology and exhibit similar tunnel-like crevices connecting the active site to the surface in their deduced 3D structures. Notably, their intermediate ancestors 96 and 132 also formed such tunnel-like cracks, but they were either narrow or had a depth of half of that of extant putative [NiFeSe] Hases. If acquisition of the Sec residue conferred higher oxygen tolerance through a lower redox potential than the sulphur counterpart, the addition of this residue may have allowed the generation of a wide-open cavity, which may have conferred a competitive advantage in processing H_2_ molecules over an undeveloped cavity, particularly when the host microbes diverged under absolutely anaerobic environments, such as deep-sea hydrothermal vents, which prevents exposure to even low oxygen levels.

Regarding the human faeces-derived SRB strains *B. wadsworthia* 3_1_6 and *D. piger*, two of the predicted structures have poorly developed gas cavities and their active sites are sealed within the protein interior. This was interpreted along with consideration of the oxidative stress under the physiological and pathological conditions possibly imposed by their intestinal habitats^15^,^16^ as natural selection against opening the gas cavity even after the acquisition of Sec, exemplifying good contrast to the close assemblages of *D. alaskensis* G20 and two *D. vulgaris* strains.

Similarly, the ternary structures predicted for the [NiFeSe] Hases from *D. postgatei* 2ac9, *D. autotrophicum* HRM2 and *D. phosphitoxidans* are obviously closed structures with small inner cavities that imperfectly connect the interior and exterior environments. The phylogenetic tree suggests that these non-iron-corrosive SRBs diverged from the absolute anaerobes at an early stage of their evolution. The sediment-derived SRB species can adapt to microbial mats that include photosynthetic, oxygen-generating, aquatic organisms and even reduce oxygen as a protective measure^17^. Sediment inhabitants encounter dynamic changes of oxic/anoxic interfaces daily in the photonic zones of microbial mats^18^. The cavity-less [NiFeSe] Hase may be the consequence of molecular evolution to adapt to environments where oxygen-saturated conditions can occur upon exposure to sunlight.

## Discussion

The phylogenetic tree of [NiFe] and [NiFeSe] Hases strongly implied conceivable evolutionary traits of [NiFeSe] Hases, which may have evolved through mutation and gene duplication of a more ancient [NiFe] Hase at an early stage of molecular evolution. The in-frame opal codon UGA can occur randomly from a Cys-coding triplet (UGC or UGU) by a single substitution, but selenoprotein biosynthesis requires functional selenosome machinery. Presumably, the co-translation of UGA as a Sec residue was already in practice for decoding other Se-containing proteins, such as formate dehydrogenases, when [NiFeSe] Hase evolved upon the mutation of Cys to Sec.

**Figure 6:**
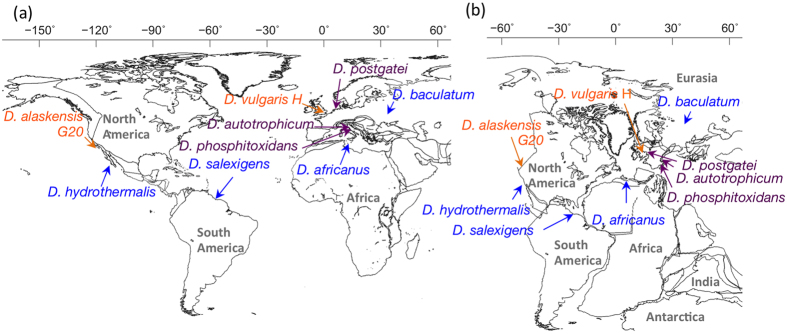
Geographic positions where the SRB strains were isolated are located on the current world map (**a**) and on the PANGEA continent 200 Mya (**b**). Maps were created with GPlates visualisation software as described previously^36^.

The phylogenetic retrospection suggesting that the putative and established Sec-containing Hases diverged from a common ancestor questions the wide distribution of these anaerobic bacteria (Fig. 6a). Three closely related species, *D. vulgaris* Hildenborough, *D. vulgaris* Miyazaki F and *D. alaskensis* G20, were isolated from soil in Hildenborough, England, a paddy field in Japan, and Venture County, CA, USA, respectively. Another tight clade, implying a long-distance habitat range, extends from the Libyan sea (*D. africanus* Walvis Bay from the submarine H_2_S eruption spot) to mud in British Guyana in South America (*D. salexigens* DSM 2638) and a hydrothermal chimney wall in the East Pacific Rise (*D. hydrothermalis* DSM 14728).

Such global distribution may be attributable to the antiquity of host microbes growing by sulphate-dependent respiration. The ~3.47-gigayear (Gy)-old sulphur-isotope record of sedimentary barites from North Pole, Western Australia, provides the oldest evidence that mesothermic, dissimilatory SRB participated in the geographic sulphur turnover^19^. Among bacteria, the major sulphate reducers are mesophiles, including the Firmicutes, Nitrospirae and δ-subdivision of the Proteobacteria, which included archetypal sulphate-reducing *Desulfovibrio* species that are estimated to have diverged 2.2 Gy ago according to phylogenetic analysis^20^. Thus, the phylogeny of [NiFeSe] Hase may provide an intriguing evolutionary model, in which molecular evolution and possible functional diversification behind the structural change may be linked to diastrophism of the Earth and consequent environmental changes.

Ancestral species of SRB must have resided on the Earth and survived several mass extinction events, including the greatest biotic crisis and superanoxic period at the Permian–Triassic transition 252 million years ago (Mya)^21^. Accordingly, the isolation spots of extant [NiFeSe] Hase producers can be linked to the corresponding areas on the Pangea supercontinent 200 Mya^22^, where close assemblages can be identified as closer residents (Fig. 6b). The global expansion of hosts harbouring [NiFeSe] Hases can be understood by assuming that continental drift extended the habitat range of the host bacteria across the Atlantic Ocean and beyond Europe. Alternatively, the geographical patterns of these anaerobic SRB may be attributable to other possibilities, including carriage by macro-organisms, such as fish and birds, long-range airborne particles across continents^23^ or possibly the consequence of the old microbial tenet, ‘everything is everywhere, but environment selects’^24^. Although it remains unclear how the common ancestral SRB harbouring the whole set of genes for [NiFe]/[NiFeSe] Hases were globally dispersed, the molecular evolution of a gas cavity appears to be driven mainly by the environmental factors of hydrogen availability and oxygenation risk.

**Figure 7:**
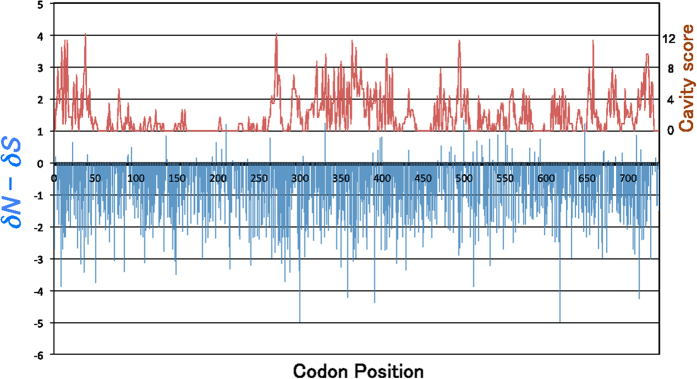
Synonymous and non-synonymous mutations along the aligned codons for the small subunit (1–361) and large subunit (362–777). Codons with more non-synonymous substitutions (*δN* − *δS* > 0) are positive in the blue bars. Negative bars implicate highly conserved sites that accumulated synonymous mutations. The red graph of cavity scores represents the number of amino acid residues involved in cavity formation.

To investigate the mechanism of cavity formation, the numbers of amino acid replacements *versus* silent mutations were compared at each position in the aligned sequences of the ancestral and extant [NiFeSe] Hases. The number of non-synonymous mutations per replacement site was termed *δN* and that of synonymous mutations per silent site was termed *δS*. The intensity of positive selection, reflected by the magnitude of *δN* − *δS*, was overlaid with the number of residues involved in the cavity formation (Fig. 7). Accordingly, cavity-forming residues were identified even among highly conserved residues, for which *δN* − *δS* < 0. Remarkably, positions that have accumulated non-synonymous mutations accounted for only 39 of 777 aligned codons (Supplementary Fig. S9), suggesting that only 5% of the amino acid residues dominate the subtle conformational change of the remaining 95% of highly conserved residues, for which *δN − δS* < 0.

**Figure 8:**
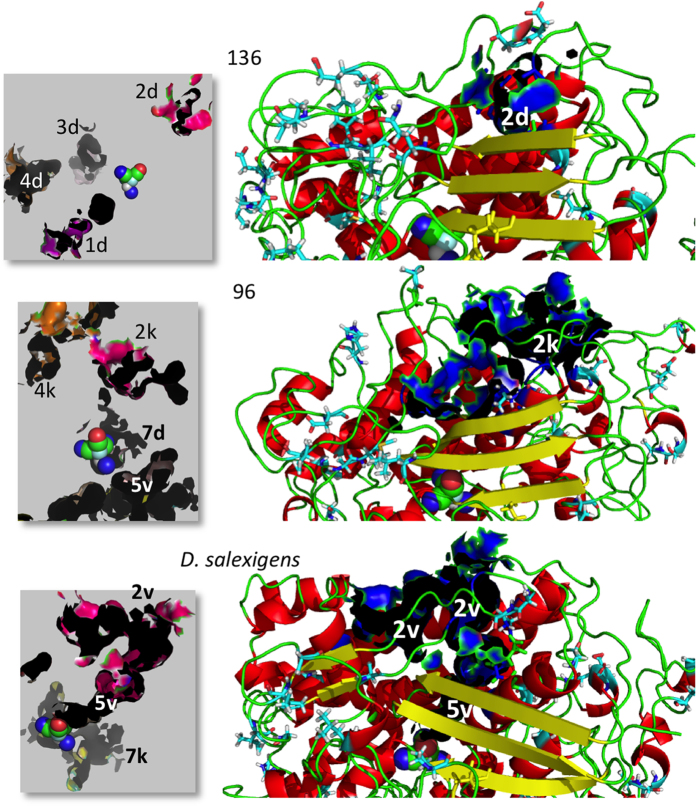
Regional conformation changes during cavity development from ancestral 136 to *D. salexigens* [NiFeSe] Hase. The development of cavity #2 is shown in a close-up view. The amino acid residues that have changed through non-synonymous mutation (*δN* > *δS*) are depicted as sticks, whereas highly conserved residues (*δS* > *δN*) are shown as cartoon representation. Left panels represent cavities around the Ni-Fe active site.

An example of progressive development of cavity #2 is shown in a close-up view to illustrate the regional conformational change of the main chain (Fig. 8). It was evident that gas cavities evolved through regional conformational changes in the polypeptide chains. These changes appear to be underpinned by the substitution of several key residues in the vicinity and/or at remote positions.

## Methods

### Phylogenetic analysis

The evolutionary traits of the [NiFeSe] Hases are based on the phylogenetic analysis of 88 microbial genomic sequences, including those from the genomes of 76 δ-Proteobacteria and 11 Clostridia, as well as those of the *Escherichia coli hyaAB* genes. The nucleotide sequences of the small and large subunits were combined into a single assemblage. Multiple alignments of these coding sequences were constructed using MUSCLE^11^ and further refined using the PRANK algorithm in GUIDANCE^12^. An ML phylogenetic tree was constructed from this alignment using a general time-reversible model with G + I evolution rates, which was considered the best model for the evolution of the subject DNA sequences. The reliability of the inferred tree was assessed by 300 repetitions of the bootstrap test, conducted using MEGA 5.2.2 software^13^. Ancestral sequences were reconstructed based on the phylogenetic tree and then a codon-based positive selection test was conducted using the Nei-Gojobori model^25^.

### Pairwise alignment and homology modeling

Obtaining a high-quality 3D homology model of a target protein essentially depends upon high-quality alignment in the pairwise comparative model. Misallocation of gaps in the amino acid sequence alignment of the target and template sequences sometimes occurs when using conventional threading methods, which causes the modelling process to set imprecise boundaries for the different secondary structural elements of the target sequence. On the other hand, the sequences encoding the interior sections of a protein tend to be more conserved than those creating cavities on the protein surface. Consequently, residues from surface regions may be less reliably aligned than those from interior regions. We used the AladeGap algorithm for sequence alignment in the present study. The protocol considers the characteristics of protein evolution in which insertion and deletion of residues occurs more frequently on the surface than in the interiors of secondary structures tightly folded into helices and strands^26^. The resulting alignment was used to build the ternary folds of polypeptide chains using the SWISS MODEL web server in alignment mode^27^. The polypeptide scaffolds were then joined with atomic coordinates for metallic ligands, which are connected to Cys, Sec and His amino acid residues. The resulting geometry was further optimised using the Molecular Operating Environment (MOE) platform (Chemical Computing Group, Inc., Montreal, Canada). During this process, the interior networks of van der Waals interactions, charge–charge interactions, hydrogen bonding and tautomer orientation were optimised using a protonate 3D matrix^28^ in the implicit Born solvent model^29^.

### MD simulation

The model structure obtained on the MOE platform was further improved by performing a MD simulation using the AMBER11 program supplemented with density functional theory-based ensemble parameters^30^ for the metallic, redox-active ligands of the [NiFe] Hases in the isotherm–isobar system. Additional parameters for Sec were set by modifying the corresponding sulphur parameters, considering both the longer bonding distance (Se: 190 pm versus S: 180 pm in VdW radius) and an average atomic mass of 78.96. Thus, the protein structure was first relaxed by MD simulation with the Generalized Born solvation model for a few nanoseconds. This was followed by insertion of the atomic coordinates into a cubic box, with a minimum distance of 12 Å between the protein and the side of the box, filled with TIP3P water molecules^31^. The aqueous protein system was neutralised by adding sodium cations at the protein’s minimum electrostatic potential. Simulations were conducted in the PME-MD-MPI module using the SHAKE algorithm on bonds involving hydrogen atoms. Annealing simulations for 1-ns intervals were conducted by creating 500,000 consecutive frames at 2-fs increments, and one of every 50 frames was picked up and aligned to make a trajectory file for a 1-ns time interval. Normally, we could gain an initial equilibrated conformation in the first 3–4 ns; then, the model reached a satisfactorily equilibrated conformation by further optimisation up to 11 ns. The average atomic coordinates were computed from the trajectory of 10,000 frames created by the MD calculations from 10 to 11 ns. The stereochemistry of the resulting model was tested using the website ProSA-web^32^, and the structure was submitted to CASTp calculations for identification of gas cavities.

### Cavity identification

CASTp was used to characterize the surface features of the ancestral and extant putative [NiFeSe] Hase structures annealed by MD simulation. Gas channels and insulated internal cavities were identified using a geometric computation method^33^,^34^,^35^. The cavities identified using CASTp calculations were depicted using the PDB viewer PyMOL, implemented with a CASTp plugin. Amino acid residues involved in cavity formation were also identified using the PyMOL viewer and aligned on a *δS*-versus-*δN* plot on the basis of a codon-based Z-test performed using MEGA 5.2.2 software.

## Additional Information

**How to cite this article**: Tamura, T. *et al.* Molecular Evolution of Gas Cavity in [NiFeSe] Hydrogenases Resurrected *in silico*. *Sci. Rep.* **6**, 19742; doi: 10.1038/srep19742 (2016).

## Supplementary Material

Supplementary Information

## Figures and Tables

**Figure 1:**
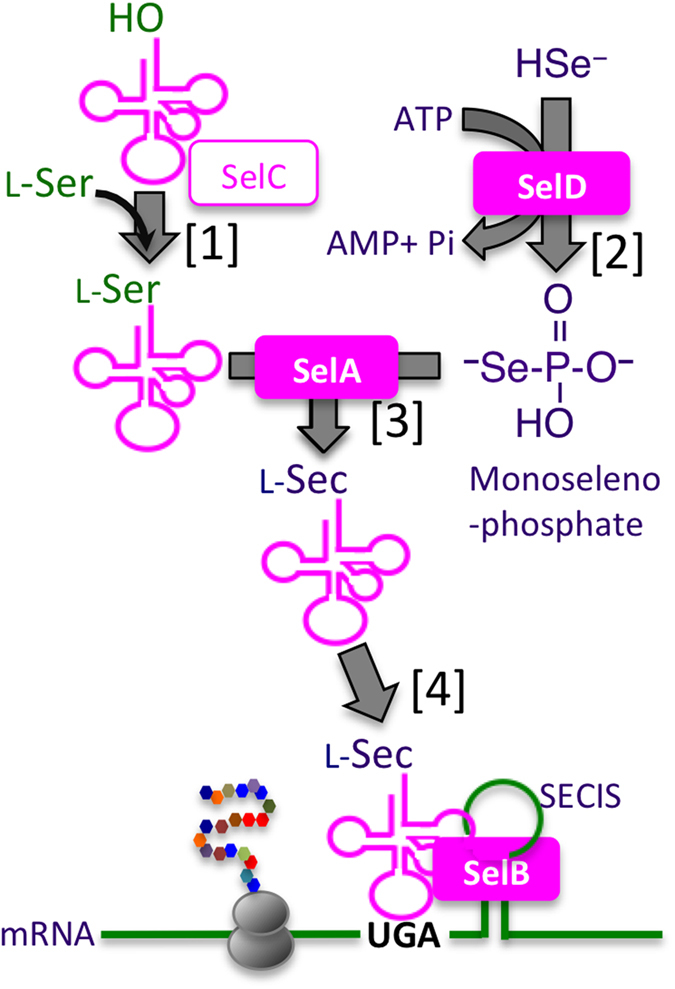
Decoding an in-frame opal codon (UGA) by selenosome. Sec is directed by the in-frame opal codon (UGA), and is synthesized on a specific tRNA^UGA^ (*selC* product) that is initially charged with L-serine [**1**]. A reactive selenium donor compound, monoselenophosphate, is synthesized from ATP and selenide by the catalysis of selenophosphate synthetase, the *selD* product [**2**]. L-Sec-tRNA^UGA^ is synthesized by L-selenocysteine synthase, *selA* product, which converts L-Ser to L-Sec on the tRNA^UGA^ [**3**]. SelB is an elongation factor, which binds to L-Sec-tRNA and the stem-loop structure, SECIS element, designating the position to incorporate L-Sec in a growing polypeptide chain [**4**].

**Table 1:**
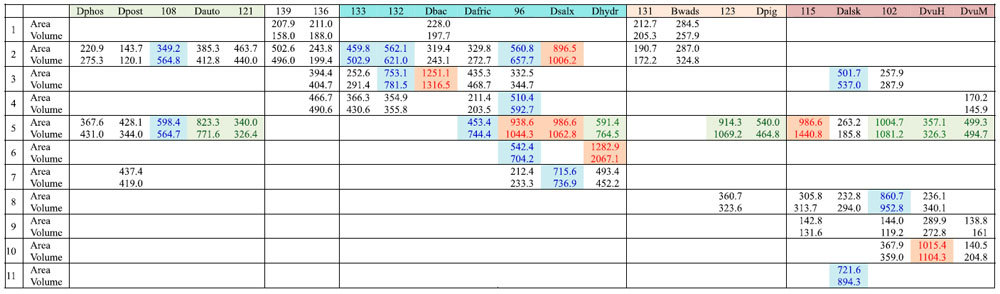
Area and volume of cavities identified in ancestral and extant [NiFeSe] Hases.

Based on the volume (Å^3^), the cavities are classified as dent (volume < 500 Å^3^), crack (blue: 500 Å^3^ ≦ volume < 1000 Å^3^), or crevice (red: 1000 Å^3^ ≦ volume). The internal cavity is designated in green. Abbreviations used are Dphos (*Desulfotignum phosphitoxidans*), Dpost (*Desulfobacter postgatei* 2ac9), Dauto (*Desulfobacterium autotrophicum* HRM2), Dbac (*Desulfomicrobium baculatum* DSM 4028), Dafric (*Desulfovibrio africanus* Walvis Bay), Dsalx (*Desulfovibrio salexigens* DSM 2638), Dhydr (*Desulfovibrio hydrothermalis* DSM 14728), Bwads (*Bilophila wadsworthia* 3_1_6), Dpig (*Desulfovibrio piger* ATCC 29098), Dalsk (*Desulfovibrio alaskensis* G20), DvuH (*Desulfovibrio vulgaris* Hildenborough), and DvuM (*Desulfovibrio vulgaris* Miyazaki F).
