# Supplementary material for: Molecular evolution of gas cavity in [NiFeSe] hydrogenases resurrected in silico
Source: Sci Rep. 2016 Jan 28;6:19742. doi: 10.1038/srep19742 (PMC4730141; doi:10.1038/srep19742)
Supplement: Supplementary Information [file srep19742-s1.pdf]

# Supplementary materials

## Title: Molecular evolution of gas cavity in [NiFeSe] hydrogenases resurrected *in silico*

**Authors:** Takashi Tamura[1,2,3]*, Naoki Tsunekawa[2], Michiko Nemoto[1], Kenji Inagaki[1], Toshiyuki Hirano[2], Fumitoshi Sato[2]

**Affiliations:**

[1]Graduate School of Life and Environmental Science, Okayama University, Okayama, 700-8530, Japan.

[2]Institute of Industrial Science, the University of Tokyo, Komaba 153-8505, Japan.

[3]PRESTO, Japan Science and Technology Agency.

*Correspondence to: tktamura@okayama-u.ac.jp.

**This PDF file includes:**

supplementary Figures S1 to S9

supplementary  Table S1

Supplementary Fig. S1

>*D. vulgaris* Hildenborough

GACCCGTGActgggctgtgccgtgcacgtgctgcacgctgagtccggcaaggtcgccgtcatcg
aagtgaagtag

ΔG = -27.70 kJ/mol

```
          10        20
  |   CG  A       gu  c     cg
--GACC  UG cugggcu  gc gugca  \
  cugg  ac ggccuga  cg cacgu  u
\ ^   a-  -       gu  -     cg
     50          40         30
       60
  gccg-    c
       ucau g
       agug a
  gauga    a
      70
```

>*D. vulgaris* Miyazaki F

GACCCGTGActgggctgtgccgtgcacgtgctgcacgctgaatccggcaaggtctccgtcgtcg
aagtgaagtag

ΔG = -22.80 kJ/mol

```
                10         20           30
GACC-------  -    u-      g     ug--  -|   c
           CG UGAc  gggcu ugccg    ca cgug \
           gc gcug  ucugg acggc    gu gcac u
gaugaagugaa  u    cc      a     cuaa  c^   g
    70        60         50        40
```

>*D. alaskensis* G20

GACCCGTGActgggctgcgccgtgcacgtgctgcacgcagagtccggtaaggtttccgtagtcg
aattctag

ΔG = -23.90 kJ/mol

```
                          10        20
---------   --------| GUG        gc  c     cg
         GAC        CC   Acugggcu  gc gugca  \
         cug        gg   uggccuga  cg cacgu  u
gaucuuaag   augccuuu^ aa-        ga  -     cg
 70        60        50         40         30
```

Supplementary Fig. S1

>*D. piger*

GACCCGTGActgggctgtgccgtgcacgtgctgcacgctgagaccggcgaagaatccatcgtga
acctgggctag

ΔG = -20.80 kJ/mol

```
            10        20
GA--   .-GUGA| g   u    c   c
    CCC      cu ggc gugc gug a
    ggg      ga ucg cacg cgu c
gauc   \ ----^ g   -    u   g
    70        40         30
                 50
         accg--    aga
               gcga   a
               ugcu   u
         uccaag    acc
                60
```

> *B. wadsworthia* 3-1-6

GACCCGTGActgggttgtgccgtgcacgtgctgcacgccgaatccggcaaggttgccgtcatca
atatggattaa

ΔG = -23.50 kJ/mol

```
          10        20
  |   CG  A       g  c      cg
--GACC  UG cuggguu ug cgugca  \
  uugg  ac ggccuaa gc gcacgu  u
\ ^   a-  -       -  c      cg
     50           40        30
          60
  g----    cau
     ccgu   \
     ggua   c
  aauua    uaa
      70
```

>*D. baculatum*

GACCCGTGActgggctgtgccgtgcacgtgctgcacgctgagaccggtgaagaacacgttgtca
acattgactaa

ΔG = -21.50 kJ/mol

```
                           10        20
------------   C-    ------|    g  gu  c     cg
            GAC  CGUG      Acugg cu  gc gugca  \
            cug  gcac      uggcc ga  cg cacgu  u
aaucaguuacaa   uu    aagaag^    a  gu  -     cg
    70        60         50         40         30
```

Supplementary Fig. S1

>*D. salexigens*

GACCCGTGActgggttgtgccgtgcacgtgctgcacgcagagaccggtgaagaacatgttgttc
acgtaggcgaaggttgctaa

ΔG = -26.80 kJ/mol

```
        10          20
GACCC UG   .-g    g-|  c     cg
     G  Acu   gguu  ugc gugca  \
     c  ugg   ccag  acg cacgu  u
aau-- gu   \ -    ag^  -     cg
     80           40        30
                        50
             g--------     g  c
                      gugaa aa a
                      cacuu uu u
             aagcggaug     g  g
                 70        60
```

>*D. africanus* Walvis Bay

GATCCGTGActgggttgcgccgtgcacgtgttgcacgctgagactggcgaaaagaccgttgtca
ctgtcgagtag

ΔG = -22.40 kJ/mol

```
              10            20           30
------   CC     ug-    g---    -  g-  -|   u
      GAU  GUGAc   gguu    cgcc gu  ca cgug \
      cug  cacug   ccag    gcgg ca  gu gcac u
gaugag   u-     uug    aaaa    u  ga  c^   g
    70         60         50         40
```

>*D. hydrothermalis*

GATCCATGActgggctgtgccgtgcacgtgctgcacgcagagaccggtgaagagcatgtttacc
acgtgggagaaggctgctag

ΔG = -26.40 kJ/mol

```
          10        20
.-GAUCCAUG|    g  g   c     cg
          Acugg cu ugc gugca  \
          uggcc ga acg cacgu  u
\ --------^    a  g   -     cg
                 40         30
  50         60
  ga  -   aug   a  a
    ag agc   uuu cc c
    uc ucg   aga gg g
  ga  g   ga-   g  u
     80          70
```

>*D. postgatei* 2ac9 Supplementary Fig. S1

GACCCCTGActgggctgcgccgtccacgtgctggacgcagataccggcaagcaaatcaaagtgg
aagttcccctgtaa

$\Delta G$ = -17.10 kJ/mol

```
          10          20
.-GACCCC  A    g--|   gcc     cg
        UG cugg   cugc   gucca  \
        ac ggcc   gacg   caggu  u
\ ------  -    aua^   ---     cg
                 40           30
 50        60
  agcaaaucaa  u  aa
            ag gg  \
            uc cc  g
  aaug------  c  uu
               70
```

>*D. phosphitoxidans*

GACCCCTGActgggttgcgccgtccacgtgctggacgtagaaaccggtcggaccgttaaagtag
acgtacccttataa

$\Delta G$ = -23.00 kJ/mol

```
          10        20
.-GACCC|       guugcgc      cg
       CUGAcugg       cgucca  \
       ggcuggcc       gcaggu  u
\ -----^       aaagau-      cg
       50        40         30
                   60
  ac---------    aa
             cguu  a
             gcag  g
  aauauucccau    au
          70
```

>*D. autrotrophicum* HRM2

GACCCATGActgggctgtgccgtgcacgtgctgcacgcagagacaggcaaaacaaacgttgttg
agattccgctctag

$\Delta G$ = -18.70 kJ/mol

```
          10        20
.-GACCCA| A   gg  g   c     cg
        UG cug  cu ugc gugca  \
        ac gac  ga acg cacgu  u
\ ------^ g   a-  g   -     cg
                 40         30
 50        60
  aaacaaacguuguu   au
                gag  u
                cuc  c
  gau-----------   gc
                    70          4
```

**Supplementary Fig. S1:** Structural models of the SECIS element predicted from the sequence immediately after the in-frame opal (UGA) codon. The stabilizing energy and secondary mRNA structure were computed on the mFold web server: http://mfold.rna.albany.edu/

supplementary Fig. S2

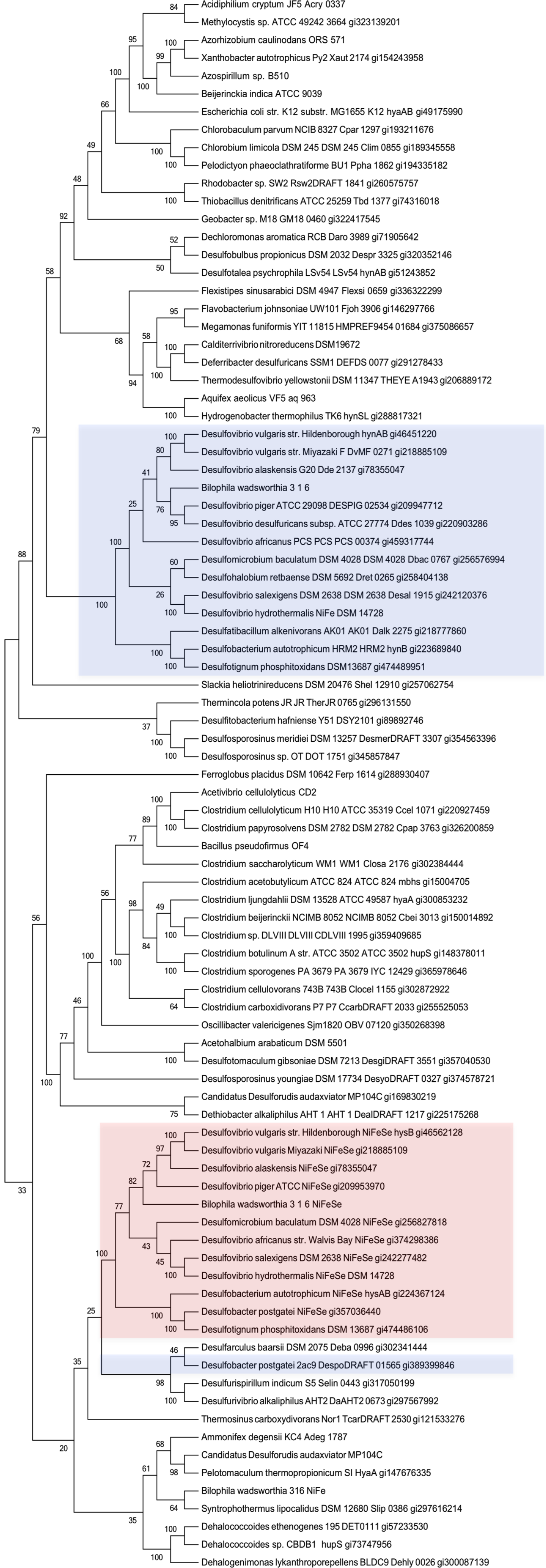


**Supplementary Fig. S2: The phylogeny of 88 [NiFe(Se)] Hases inferred by the ML method.** Below the branches are ML bootstrap percentages. Clades containing [NiFe] and [NiFeSe] Hases are shown in blue and red boxes, respectively.

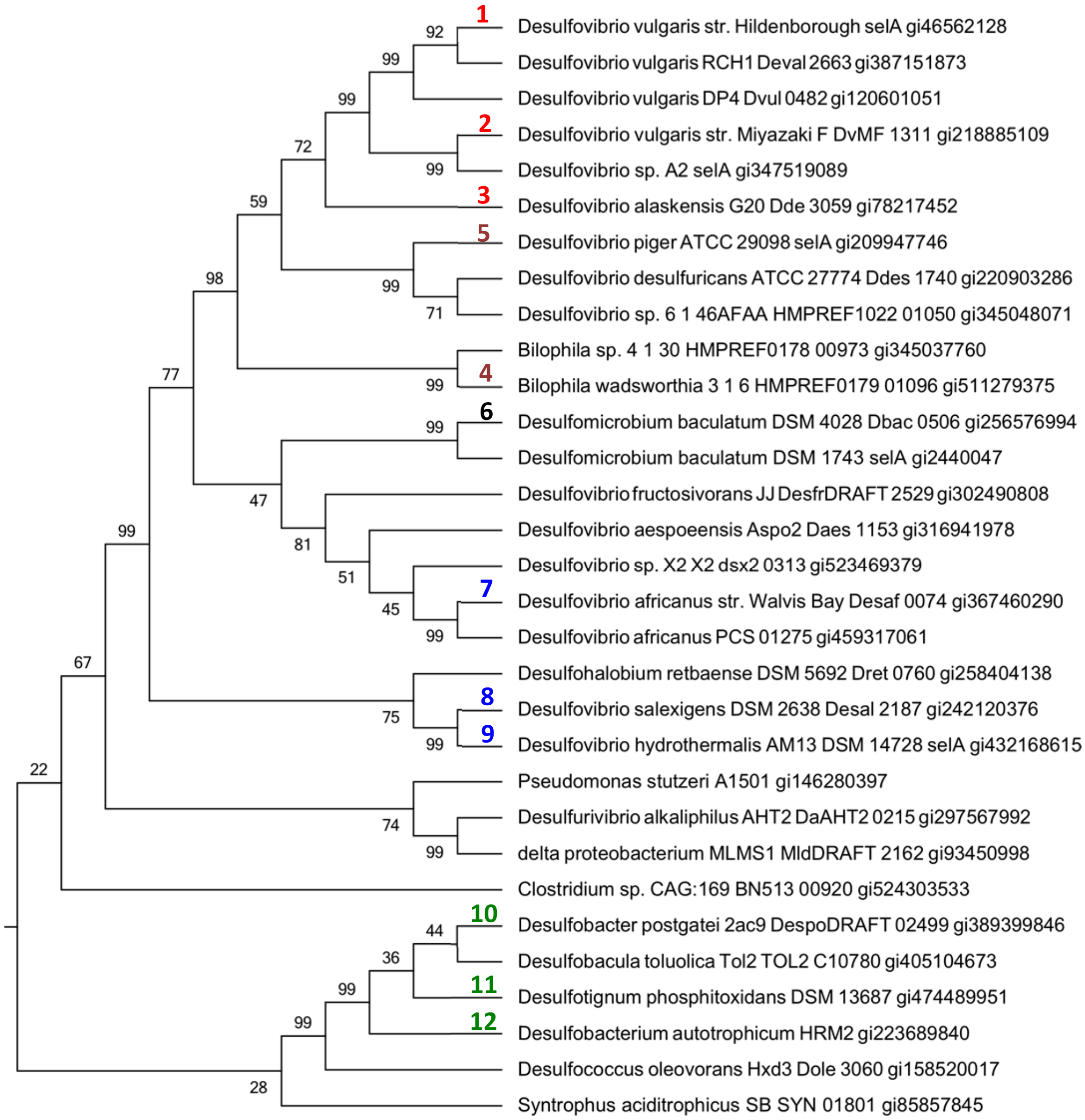


**Supplementary Fig. S3: Phylogenetic relationships of *selA* genes in a maximum-likelihood phylogenetic tree made from 74 homologous lineages.** Genes from sulphate-reducing bacteria of terrestrial origin (1-3), from clinical isolates from human faeces (4-5), of marine vent origin (7-9), and of sediment origin (10-12) are numbered in colors red, brown, blue, and green, respectively. Scores designated at each branch represent the percent of the 300-times Bootstrap test.

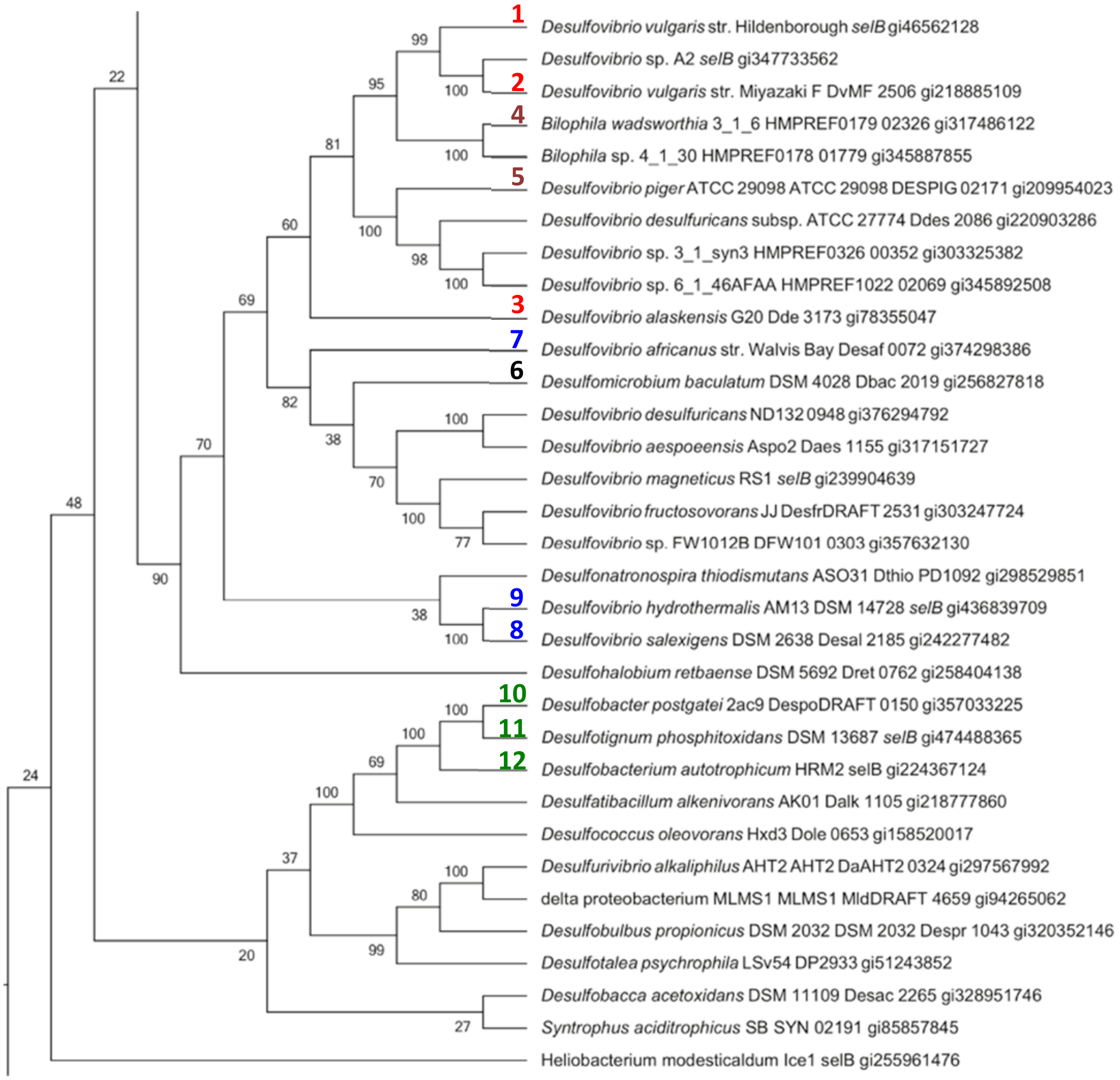


**Supplementary Fig. S4: Phylogenetic relationships among the *selB* genes in a maximum-likelihood phylogenetic tree made from 88 homologous lineages.** Genes from sulphate-reducing bacteria of terrestrial origin (1-3), from clinical isolates from human faeces (4-5), of marine vent origin (7-9), and of sediment origin (10-12) are numbered in colors red, brown, blue, and green, respectively. Scores designated at each branch represent the percent of the 300-times Bootstrap test.

Supplementary Fig. S5

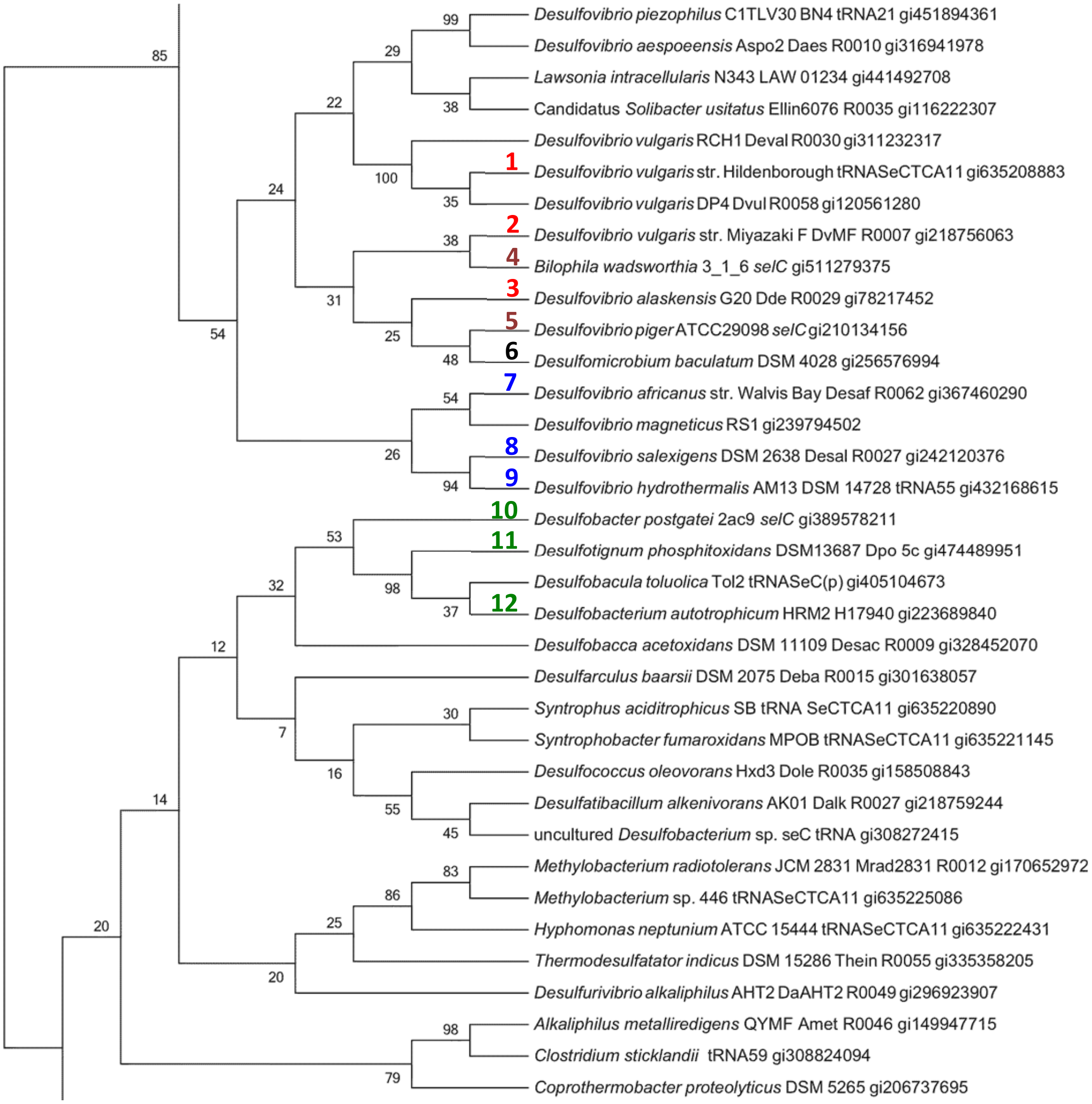


**Supplementary Fig. S5: Phylogenetic relationships of *selC* gene in the maximum-likelihood phylogenetic tree made from 48 homologous lineages.** Genes from sulphate-reducing bacteria of terrestrial origin (1-3), from clinical isolates from human feaces (4-5), of marine vent origin (7-9), and of sediment origin (10-12) are numbered in colors red, brown, blue and green, respectively. Scores designated at each branch represent the percent of the 500-times Bootstrap test. The cut-off value for the consensus tree was set at a bootstrap value of 15%. Accordingly, sequences encoding *selC* were hard to discern their phylogenetic relationships except for the three linages (10-12) due to the extra 3 bases that they have between the E and T arms of their predicted cloverleaf model.

Supplementary Fig. S6

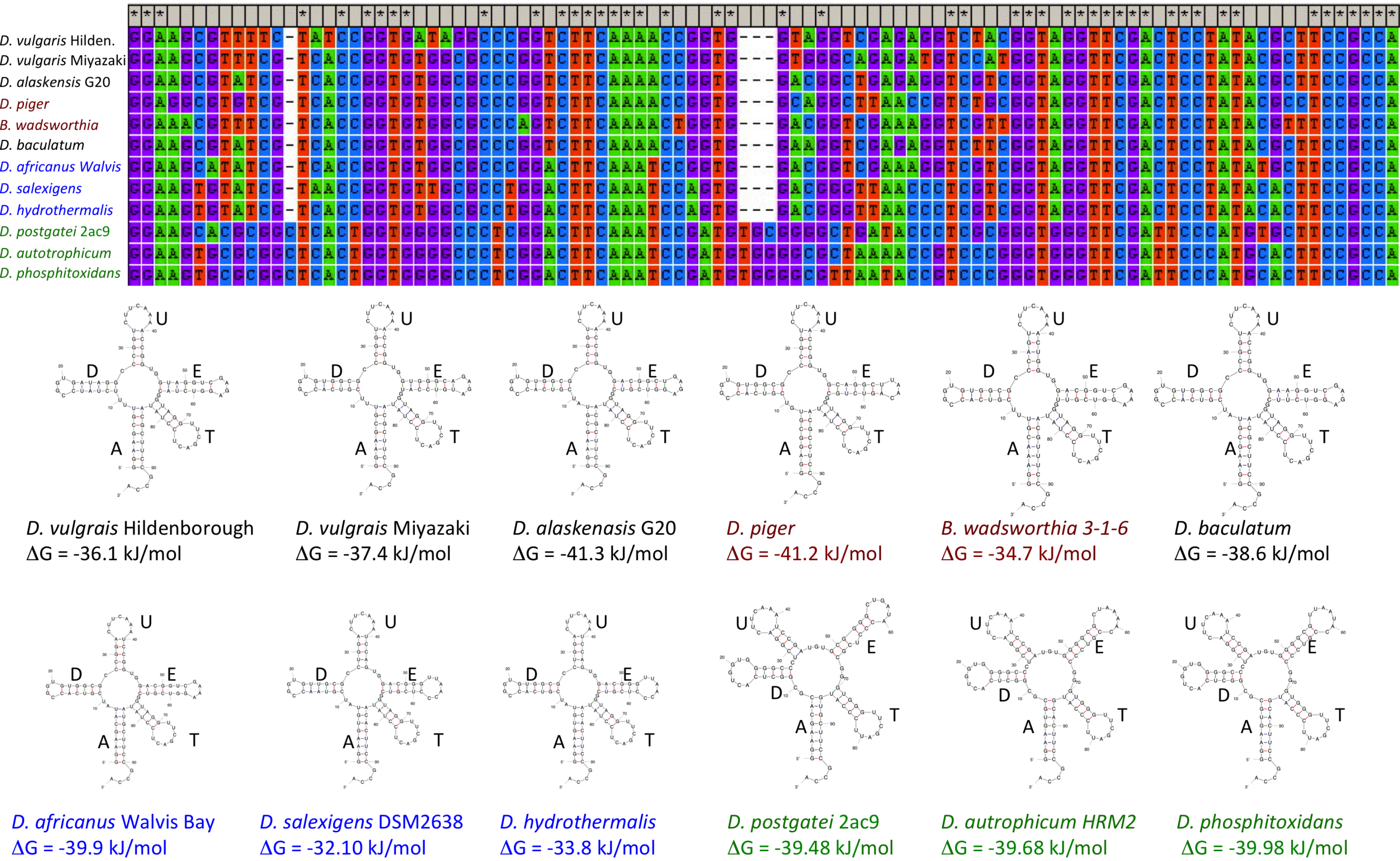


Supplementary Fig. S6: Sequence alignment and cloverleaf models for the tRNA$^{UGA}$ sequences encoded in genomes harboring [NiFeSe] Hase genes.

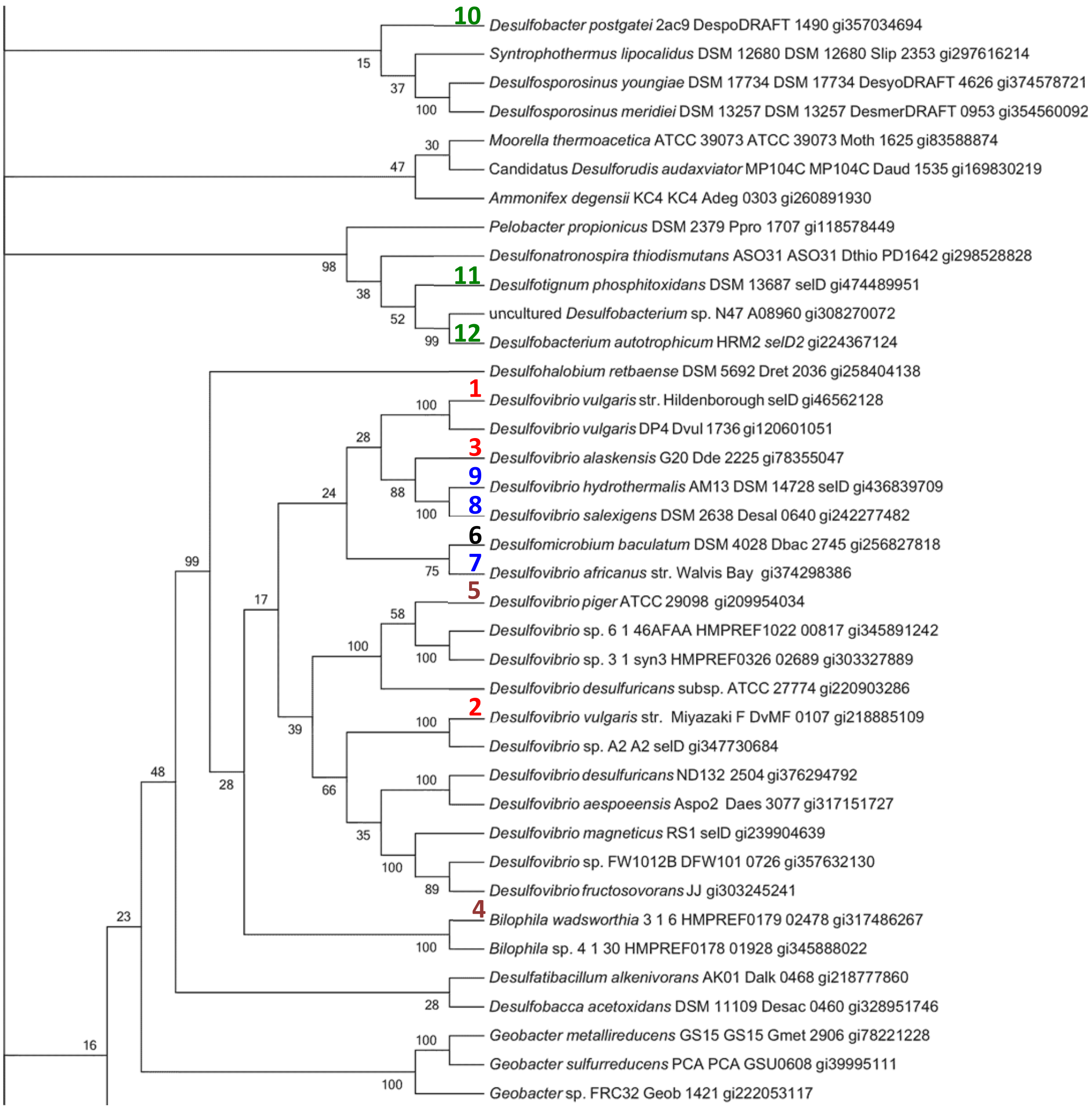


**Supplementary Fig. S7: Phylogenetic relationships of the *selD* genes in a maximum-likelihood phylogenetic tree made from 95 homologous lineages.** Genes from sulphate-reducing bacteria of terrestrial origin (1-3), from clinical isolates from human faeces (4-5), of marine vent origin (6-9), and of sediment origin (10-12) are numbered in colors red, brown, blue, and green respectively. Scores designated at each branch represent the percent of the 300-times Bootstrap test.

**Fig. S8**

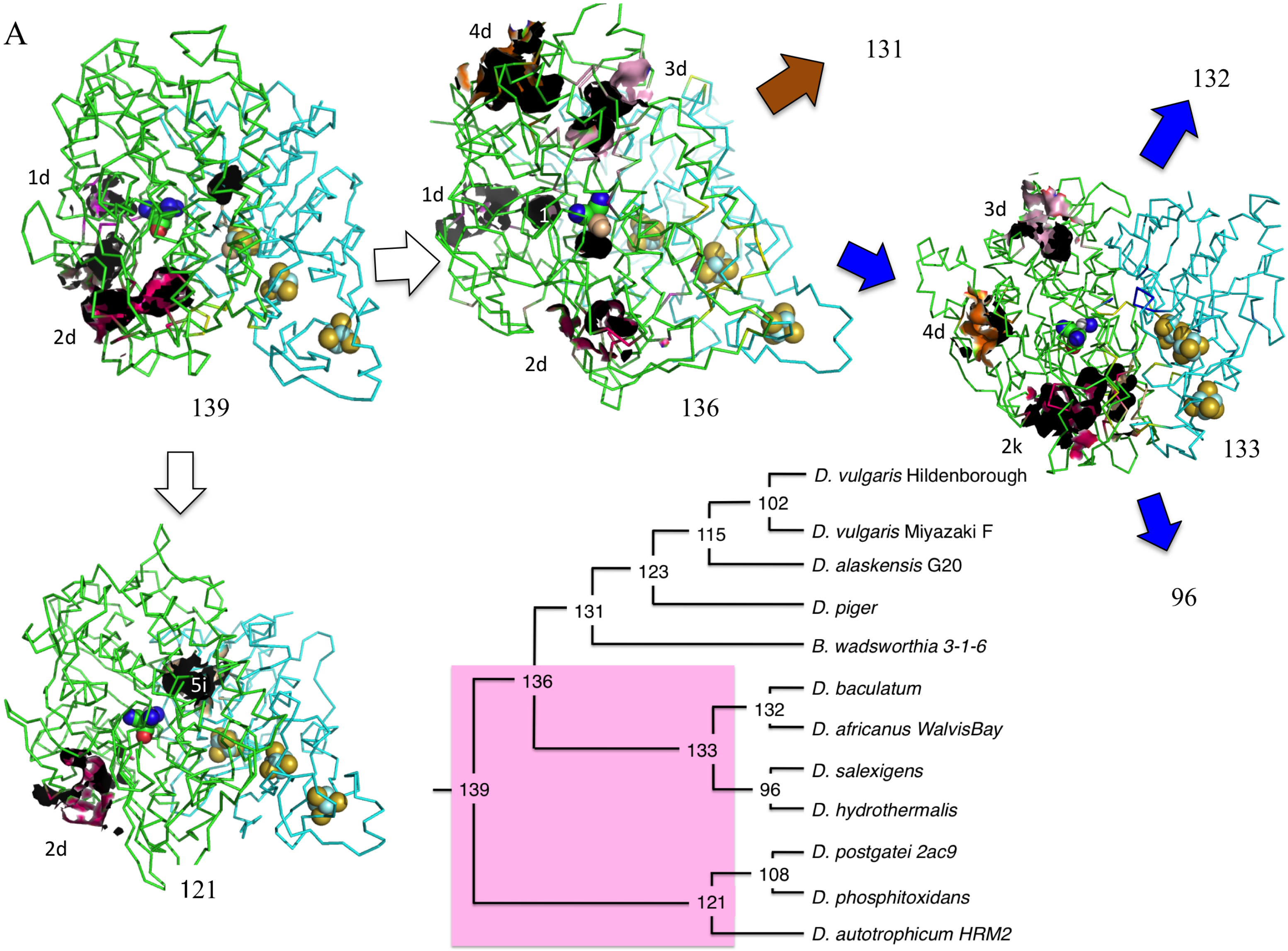
A
4d
3d
131
132
1d
1d
3d
2d
2d
4d
139
136
133
2k
96
D. vulgaris Hildenborough
102
D. vulgaris Miyazaki F
115
D. alaskensis G20
123
D. piger
131
B. wadsworthia 3-1-6
136
D. baculatum
132
D. africanus WalvisBay
133
5i
D. salexigens
139
96
D. hydrothermalis
D. postgatei 2ac9
108
D. phosphitoxidans
121
2d
121
D. autotrophicum HRM2

**Fig. S8**

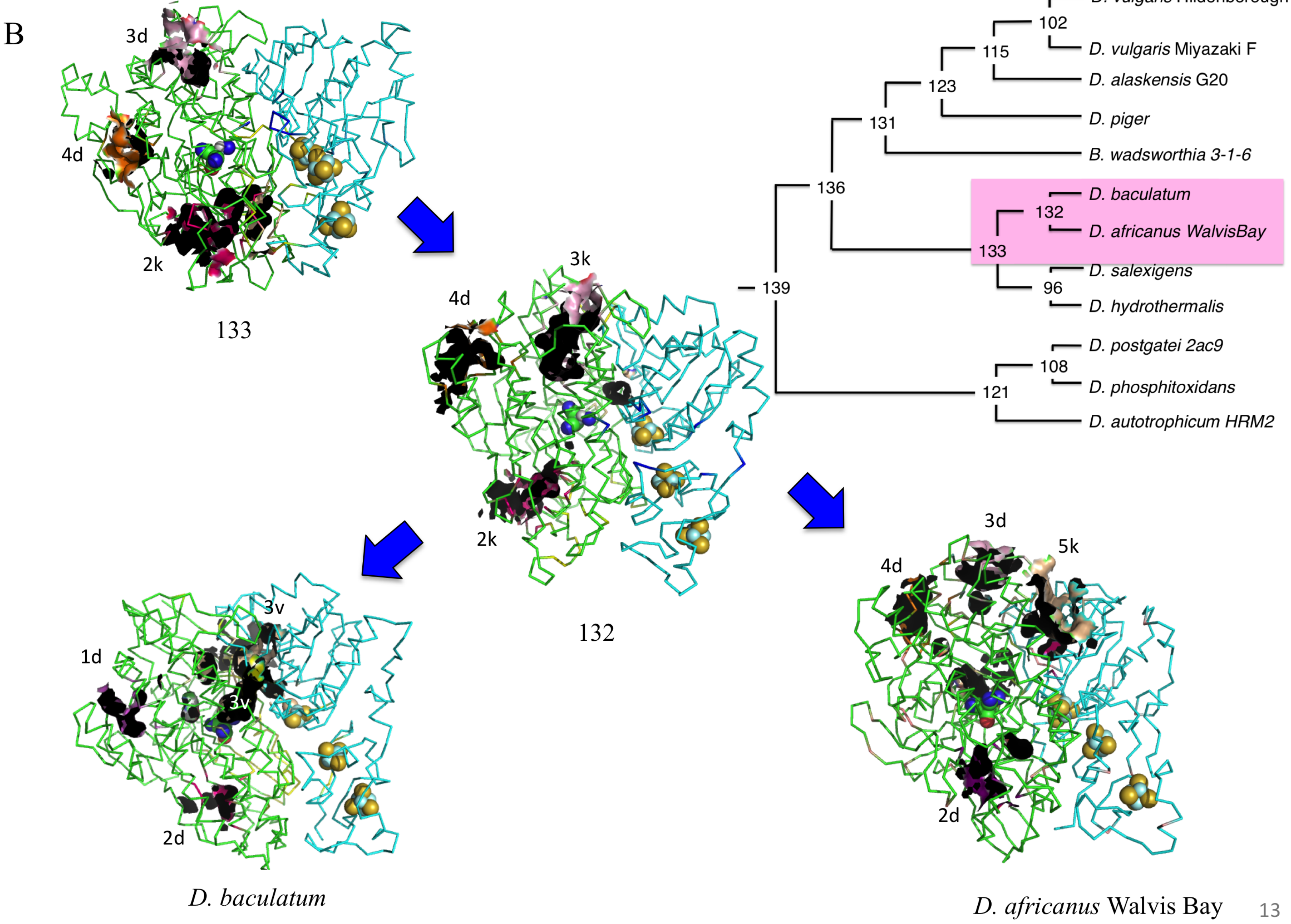
B
3d
4d
2k
133
3k
4d
2k
132
1d
3v
3v
2d
D. baculatum
3d
5k
4d
2d
D. africanus Walvis Bay
D. vulgaris Hildenborough
102
D. vulgaris Miyazaki F
115
D. alaskensis G20
123
D. piger
131
B. wadsworthia 3-1-6
136
D. baculatum
132
D. africanus WalvisBay
133
D. salexigens
139
96
D. hydrothermalis
D. postgatei 2ac9
108
D. phosphitoxidans
121
D. autotrophicum HRM2

**Fig. S8**

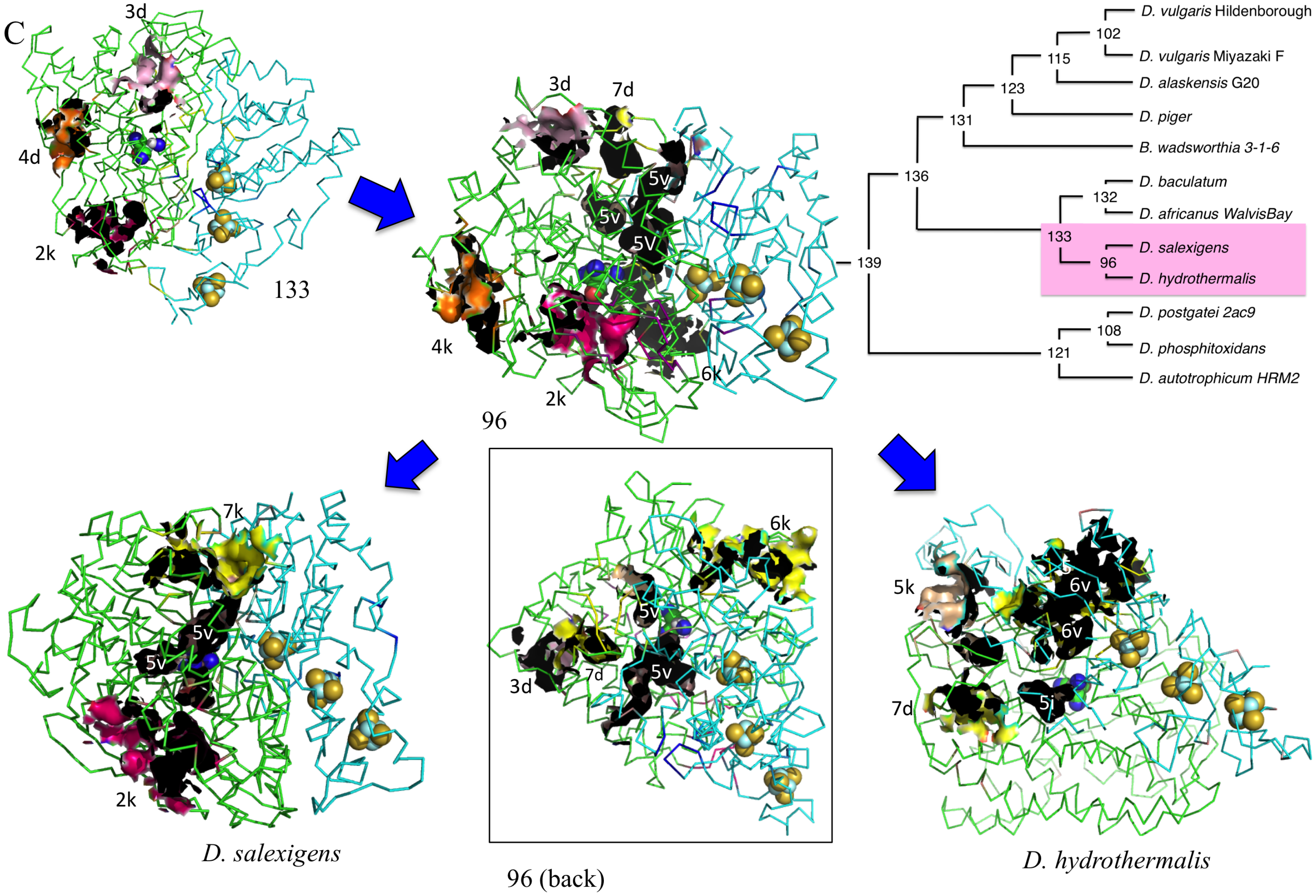
C
3d
4d
2k
133
3d
7d
5v
5v
5V
4k
2k
6k
96
D. vulgaris Hildenborough
102
D. vulgaris Miyazaki F
115
D. alaskensis G20
123
D. piger
131
B. wadsworthia 3-1-6
136
D. baculatum
132
D. africanus WalvisBay
133
D. salexigens
96
D. hydrothermalis
139
D. postgatei 2ac9
108
D. phosphitoxidans
121
D. autotrophicum HRM2
7k
5v
5v
2k
D. salexigens
6k
5v
3d
7d
5v
96 (back)
5k
6v
6v
7d
5i
D. hydrothermalis

**Fig. S8**

D

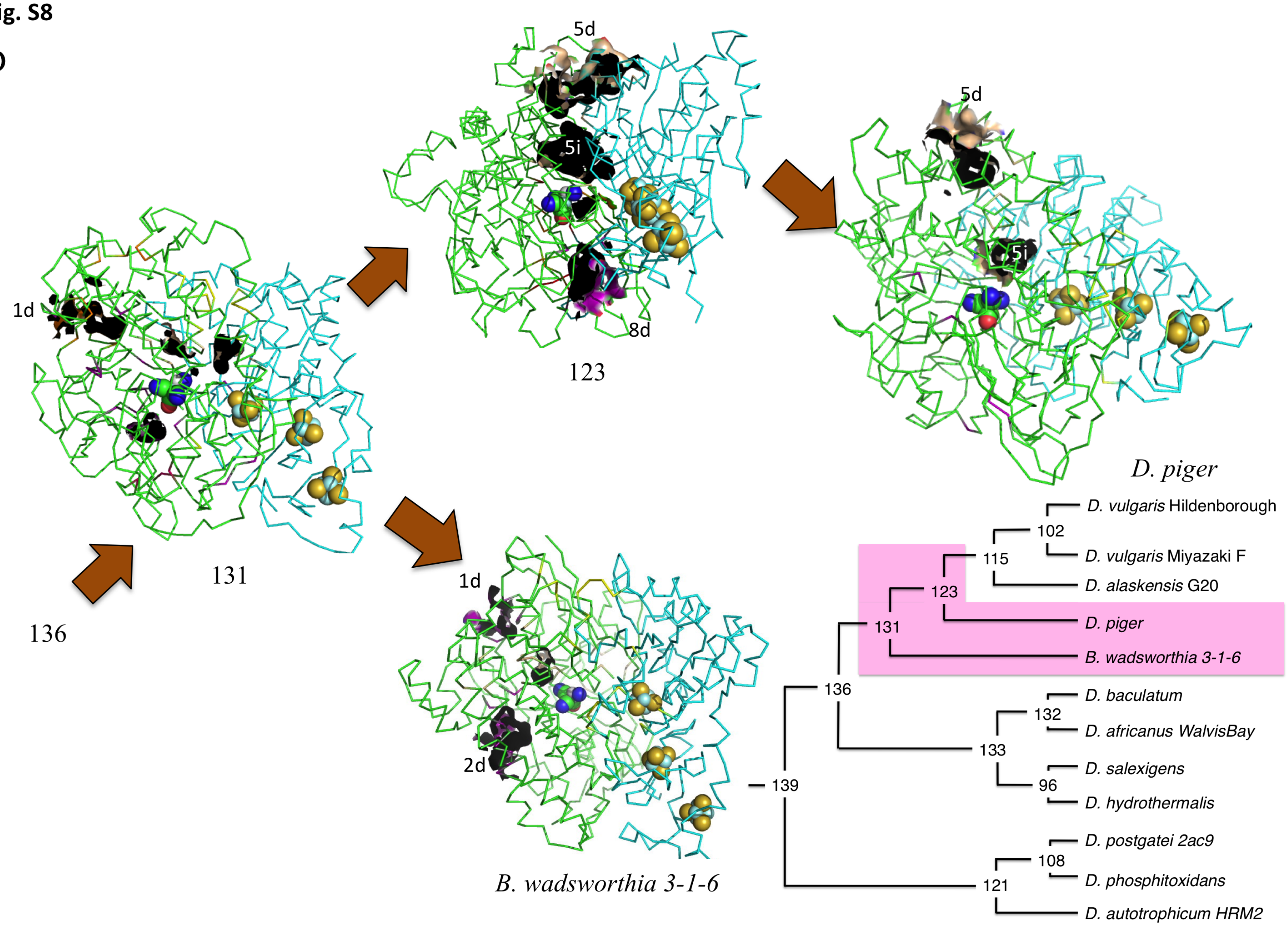
5d
5i
8d
123
5d
5i
D. piger
1d
131
136
1d
2d
B. wadsworthia 3-1-6
D. vulgaris Hildenborough
102
D. vulgaris Miyazaki F
115
D. alaskensis G20
123
D. piger
131
B. wadsworthia 3-1-6
136
D. baculatum
132
D. africanus WalvisBay
133
D. salexigens
96
D. hydrothermalis
139
D. postgatei 2ac9
108
D. phosphitoxidans
121
D. autotrophicum HRM2

**Fig. S8**

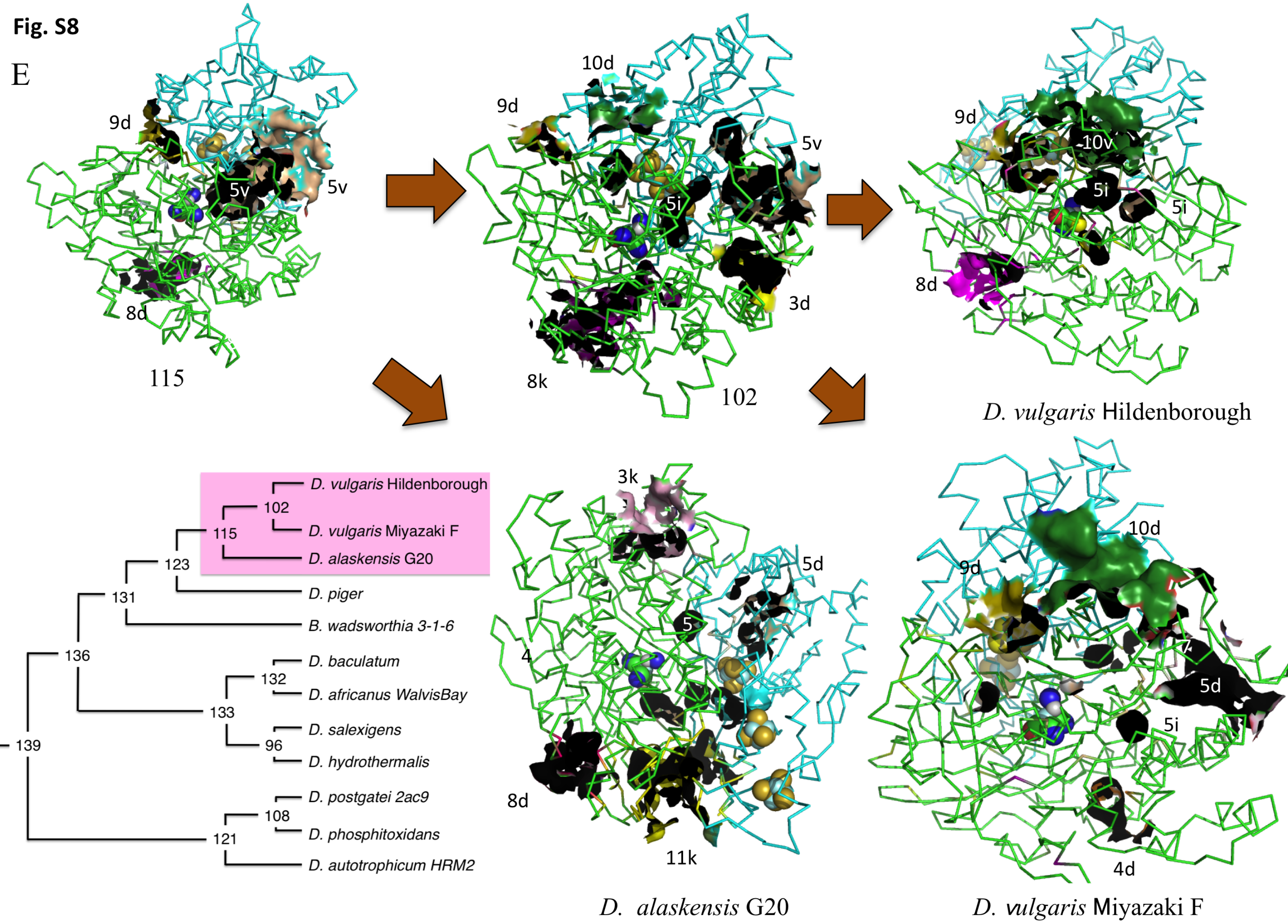
E
9d
5v
5v
8d
115
10d
9d
5v
5i
3d
8k
102
9d
10v
5i
5i
8d
D. vulgaris Hildenborough
D. vulgaris Hildenborough
102
D. vulgaris Miyazaki F
115
D. alaskensis G20
123
D. piger
131
B. wadsworthia 3-1-6
136
D. baculatum
132
D. africanus WalvisBay
133
D. salexigens
96
D. hydrothermalis
139
D. postgatei 2ac9
108
D. phosphitoxidans
121
D. autotrophicum HRM2
3k
5d
5
4
8d
11k
D. alaskensis G20
10d
9d
5d
5i
4d
D. vulgaris Miyazaki F

**Fig. S8**

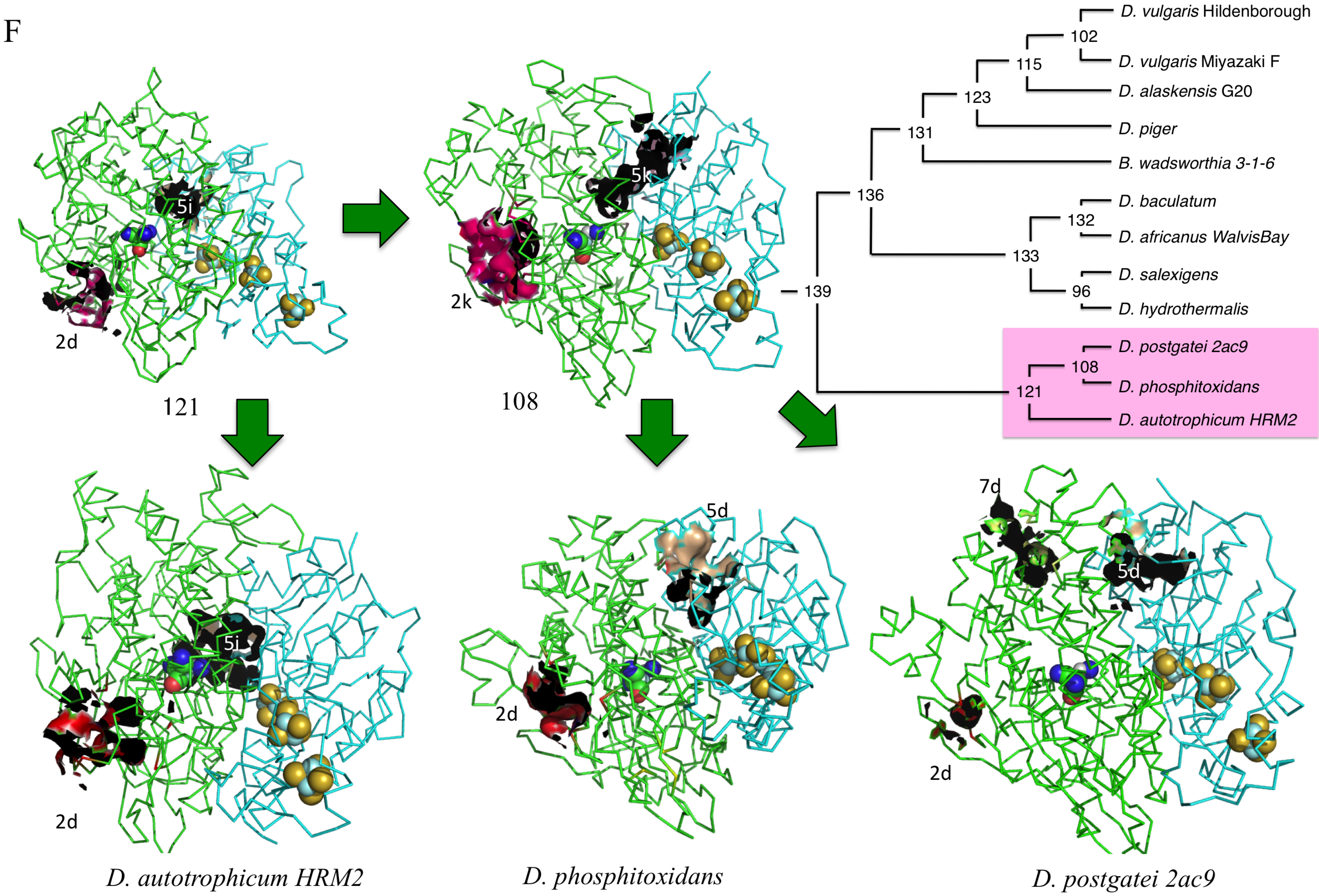
F
D. vulgaris Hildenborough
102
D. vulgaris Miyazaki F
115
D. alaskensis G20
123
D. piger
131
B. wadsworthia 3-1-6
136
D. baculatum
132
D. africanus WalvisBay
133
D. salexigens
139
96
D. hydrothermalis
D. postgatei 2ac9
108
D. phosphitoxidans
121
D. autotrophicum HRM2
5i
2d
121
5k
2k
108
5i
2d
5d
2d
7d
5d
2d
D. autotrophicum HRM2
D. phosphitoxidans
D. postgatei 2ac9

**Supplementary Figure S8: History of cavity development in [NiFeSe]Hases** Comparative morphology of the cavities in [NiFeSe] Hases from the common ancestor 136 to the extant [NiFeSe]Hases *via* the intermediate ancestral forms. Cavities are identified by location and amino acid residues involved, and are designated by numbers and morphological designations, d, k, v, and i. The Ni-Fe and Fe-S clusters are shown in sphere designation, and the large and small subunits are designated in green and blue wire forms, respectively. (A-C) Blue arrows show evolutionary trait under absolutely anaerobic aquatic environments. (D, E) Brown arrows show path to terrestrial divergence including human faeces-derived isolates. (F) Green arrows show the path to marine sediment-derived [NiFeSe] Hases. Phylogenetic tree on each panel A-F highlights the genetic trait where the panel illustrates.

**Supplementary Fig. S9**

```
                                 10        20        30        40        50        60
                         ....|....|....|....|....|....|....|....|....|....|....|....|
D. vulgaris Hilden.      ---------------------------------MSLTRRDFVKLCTGTVAGFGISQMFHP
102                      ---------------------------------MSLTRRDFVKLCTGTVAGFGISQMFHP
D. vulgaris Miyazaki F   ---------------------------------MSLNRRDFVKLCTGTVAGFGISQMFHP
115                      ---------------------------------MSLTRRDFVKLCTGTVAGFGVSQMFHP
D. alaskensis G20        ---------------------------------MSLSRRNFVKLCTGTVAGFGVSQMFHP
123                      ---------------------------------MSLTRRDFVKLCTGTVAGFGVSQMFHP
D. piger                 ---------------------------------MSLNRRDFVKLCTGTVAGFGVAQMFHP
131                      ---------------------------------MSLTRRDFVKLCTGTVAGFGVSQMFHP
B. wadsworthia 3-1-6     ---------------------------------MALSRRDFVKLCSGTVAGFGVSQMFHP
136                      ---------------------------------MSLTRRDFVKLCTGTVAGFGVSQMFHP
133                      ---------------------------------MSLTRRDFVKLCTGTVAGFGVSQMFHP
132                      ---------------------------------MSLTRRDFVKLCTGTVAGFGVSQMFHP
D. baculatum             ---------------------------------MSLSRREFVKLCSAGVAGLGISQIYHP
D. africanus WalvisBay   ---------------------------------MSLDRRDFLKLCTGTVAGLGVSQMVHP
96                       ---------------------------------MSLTRRDFVKMCTGTVAGFGISQMFNP
D. hydrothermalis        ---------------------------------MSLTRRDFVKMCTGTVAGFGISQVFNP
D. salexigens            ---------------------------------MSLTRRDFVKMCTGTVAGFGISQMFNP
121                      -------------------VKDEQTLPDAQQEGTGVSRRDFLKTLTGTAAGIGISQMFNP
D. autotrophicum HRM2    MFYCLSVTRCTSNSIKEVPLKEEQNLSDDQKEGSGVSRRGFLKALTGTAAGIGLSQMINP
D. postgatei 2ac9        -------------------VKDDHMLPETQQEKSGVTRRCFLKTVAGAAAGIGVSQMVNP
108                      -------------------VKDEQTLPDAQQEKAGVSRRNFLKTLTGTAAGIGISQMFNP
D. phosphitoxidans       -------------------VKDKQSLPDAQPKLAGISRRNFLKTLTGTAAGIGISQVFNP
139                      ---------------------------------MSLTRRDFVKLCTGTAAGFGVSQMFHP

                                 70        80        90       100       110       120
                         ....|....|....|....|....|....|....|....|....|....|....|....|
D. vulgaris Hilden.      AVHEAL-AG--T-LTGERP-PVFWLQGQGCTGCSVTLLNSVHPSIADVLLKVISLEFHPT
102                      AVREAL--G--T-LTGERP-PVFWLQG-GCTGCSVSLLNSVHP-IADVLLKVISLEFHPT
D. vulgaris Miyazaki F   AVCEAI-SG--S-LNGERP-PVLWLQGQGCTGCSVSLLNSVHPTIADVLLKVISLEFHPT
115                      AVREAL--G--T-LTGERP-PVFWLQG-GCTGCSVSLLNSVHP-IADVLLKVISLEFHPT
D. alaskensis G20        AIRDAL-AD--T-LKGDRP-PVLWLQGQGCTGCSVSLLNSVHPSIKDVLLDVISLEFHPT
123                      AIREALG----T-LTGERP-PVFWLQG-GCTGCSVSLLNSVHP-IADVLLKVISLEFHPT
D. piger                 AVREAL---AGT-LTGERP-PVIWLQGQGCTGCSVSLLNNVNPSIADVLLKIISLEYHPT
131                      AIGEAL-----T-LTGERP-PVFWLQG-GCTGCSVSLLNSVHP-IADVLLKVISLEFHPT
B. wadsworthia 3-1-6     AIHEAF---AQT-LTGERP-PVFWVQGQGCTGCSVTLLNSTHPSIADVLLKIISLEFHPT
136                      AIVEAL-----K-LAAEKP-PVFWLQG-GCTGCSVSLLNTVHP-IADVLLEVISLEFHPT
133                      AIVHAL-----KKVAAEKA-PVFWLQG-GCTGCSVSLLNTVHP-IAEVLLEVISLEFHPT
132                      AIVHAL-----KKVAAEKA-PVFWLQG-GCTGCSVSLLNTVHP-IAEVLLEVISLEFHPT
D. baculatum             GIVHAM-----T-EGAKKA-PVIWVQGQGCTGCSVSLLNAVHPRIKEILLDVISLEFHPT
D. africanus WalvisBay   AIVRAL-----E-KATSGKAPVFWLQGCGCTGCSVSILNSVHPTIADVLLKVISMDFHPT
96                       SVVHAL-----K-KFVPN---VFWLQG-GCTGCSVSILNSVHP-IAEVLLDVINLDYHPT
D. hydrothermalis        SVVHAL-----K-KFVPH---VFWLQGQGCTGCSVSILNSVHPSIAEVLLEVITLDYHPT
D. salexigens            SVVHAL-----K-KFVPN---VFWLQGQGCTGCSVSILNSVHPSIAEVLLDVINLDYHPT
121                      ALVQAL-----K-KGLEHH-PVLWIQGQGCTGCSVSLLNSVDPTIADVLLKVISLQYHPT
D. autotrophicum HRM2    ALVSAM-----K-KGLENH-PVLWIQGQGCTGCSVSLLNSVEPTIAKVLLDIISLQYHPT
D. postgatei 2ac9        TLVKAL-----E-KGLERH-PVLWIQGQACTGCSVSLLNSVDPSIADVLLKVISLQYHPT
108                      ALVQAL-----E-KGLERH-PVLWIQGQGCTGCSVSLLNSVDPTIADVLLKVISLQYHPT
D. phosphitoxidans       ALIQAL-----E-KGLERH-PVLWIQGQGCTGCSVSLLNSVDPSIADVLLKVISLQFHPT
139                      AIVEAL-----K-MAAEKP-PVFWLQG-GCTGCSVSLLNTVHP-IADVLLEVISLQFHPT
```

**Fig. S9**

```
                              130       140       150       160       170       180
                          ....|....|....|....|....|....|....|....|....|....|....|....|
D. vulgaris Hilden.       VMAWEGEHAIEHMRKVAEKFKGKFFLVIEGSVPVEADGKYCIIG-EA-----NHHEISMV
102                       -MAWEGEHAIEHMMKIAEK-KGKFFLVVEGSVPIEAD-KYCIIG-EA-----DHKEISMV
D. vulgaris Miyazaki F    VMAWEGEPAMEHMMKIAEQYKGKYFVVVEGAVPTEADGKYCIIG-EA-----HHKEISMV
115                       -MAWEGEHAIEHMMKIAEK-KGKFFLVVEGSMPIAAD-KYCIIG-EA-----DHKEISMA
D. alaskensis G20         VMAWEGEHAIEHMMKIAEKYEGKFFVVVEGSIPLAADGKYCIIG-DI-----DHKEISIA
123                       -MAGEGEDAFEHMMKIAEK-KGKFFLVVEGSIPI-ADDKYCIVG-EA-----DHKEITMA
D. piger                  VMGGEGEHDAYTHMLNIAKNFKGKFFLAIEGSIPLAKDGRYCVVA-EE-----GHTEITMA
131                       -MAAEGEDAFEHMMKIAEK-KGKFFLVVEGAIPI-ADDKYCIVG-EA-----DHKEITMT
B. wadsworthia 3-1-6      VMAAEGEGAYEHMMRVAEKFKGKFIFAVEGAVPVAHDGKCCVVA-EA-----DHHEVTMT
136                       -MAAEGEKALEHMLEIAEK-KGKFFLVVEGAIPI-ADDNYCIVG-EA-----DHKEITMT
133                       -MAAEGEKALEHMFEIAEK-KGKFFLVVEGAIPT-AEDHYCIVG-EA-----DHKEITMT
132                       -MAAEGEMALEHMFEIAEK-KGKFFLVVEGAIPT-AEDHYCIVG-EA-----DHKEITMT
D. baculatum              VMASEGEMALAHMYEIAEKFNGNFFLLVEGAIPTAKEGRYCVVG-ETLDAKGHHHEITVM
D. africanus WalvisBay    LMGAEGEMAMEHMFEVAKAKKGQYFVVLEGAIPTADE-RYCIIG-DV-----HHKEISIN
96                        -MGSEGDEAWDHMMGVAE-NKGKYILIVEGSVPTAEN-HYCIVGAGA-----DHKEYTMT
D. hydrothermalis         IMGSEGAQAWDHMMGVAGKEQGKYILIVEGSVPTAENGHYCIVGAGA-----DHKEYTMS
D. salexigens             IMGSEGHEAWDYMMSQAEANKGKYIVIVEGSVPTAENGHYCIVGAGA-----DHKEYTMT
121                       VMAGEGETALDHLYEVAEEYKGKFSLVVEGAIPMAADGKYCIVG-EN-----DHKEITMV
D. autotrophicum HRM2     VMASEGETALHNLYRVAEEYKGKFSLVVEGAIPLAANGKYCIVG-ER-----DNKDITMV
D. postgatei 2ac9         IMAGEGKTALENLFGIAREYKGKFSLVVEGAIPTAAEGKYCGVG-EY-----AHKEYTMV
108                       VMAGEGETALDHLYEVAKEYKGKFSLVVEGAIPMAADGKYCIVG-EN-----DHKEITMV
D. phosphitoxidans        VSECEGETAMNHVYEVANEYKGRFSLVIEGAIPVAADGKYCILG-EI-----GHREITMV
139                       -MAAEGEKALEHMFEIAEK-KGKFFLVVEGAIPI-ADDNYCIVG-EE-----DHKEITMT

                              190       200       210       220       230       240
                          ....|....|....|....|....|....|....|....|....|....|....|....|
D. vulgaris Hilden.       DALKEFGPNAAAVLAVGTCAAYGGIPAAEGSETGATAVSKFLGDNGIKTPVVNIPGCPPH
102                       DAIKDLAANAAAVLAVGTCAAYGGIPAAEGSETGAMA-SQFFEDNG-KTPVVNIPGCPPH
D. vulgaris Miyazaki F    DAMKSVAANAAAVLAVGTCAAYGGIPAAQGSETGAKSVSQFFKDNGIATPVVNIPGCPPH
115                       DAIKDLAANAAAVLAVGTCAAYGGIPAAEGNVTGAMA-SQFFEENG-KTPVVNIPGCPPH
D. alaskensis G20         DAMRDIAAKSAAVLAVGTCAAYGGIPAAEGNLTEAVSVQQFFEQEGIKTPVVNIPGCPPH
123                       DVIKELAANAAAVLAVGTCAAYGGIPAAEGNVTGAMG-SEFFKENG-KTPVVNIPGCPPH
D. piger                  DLVKKLAPDAAAVLALGTCAAYGGIPAAKGSVTEAMGTGALLKQAGIKTPVVNIPGCPPQ
131                       DVIKELAANAAAVLAVGTCAAYGGIPAAEGNVTGAMG-CEFFKENG-KTPVVNIPGCPPH
B. wadsworthia 3-1-6      EVTKVLAANAAAVLAVGTCAAYGGIPAGKGNETGAMGVSAFLKKEGIPAPVINIPGCPPH
136                       EVIKELATNAAAVLAVGTCAAYGGIPAAEGNLTGAMG-RDFFEENG-KTPVVNIPGCPPH
133                       EVIQELATNAAAVVAVGTCAAYGGIPAAEGNLTGSMG-RDFFEENG-KTPVVNIPGCPPH
132                       EVIQELATNAAAVVAVGTCAAYGGIPAAEGNLTGSMG-RDFFEEKG-KTPVVNIPGCPPH
D. baculatum              ELIRDLAPKSLATVAIGTCAAYGGIPAAAGNVTGSKSVRDFFAEEKIEKLLVNVPGCPPH
D. africanus WalvisBay    QAMLELGKDAAACIALGSCGAYGGIPAAKGNLTGCVGLRDFYQSKNISTPVVNIPGCPPH
96                        EATLEMAKNAAVVVNVGTCAAYGGIPAAEGNLTGSMS-TNFLAENG-KTPVVNIPGCPPH
D. hydrothermalis         EATLEMAKNAAVVVNVGTCAAYGGIPAAEGNLTGSMSVTNFLAENGVKTPVVNIPGCPPH
D. salexigens             EATLEMAKNAAVVVNVGTCAAYGGIPAAEGNLTGSMSVTNFLAENGVKTPVVNIPGCPPH
121                       DMIKELATMAGTVLAVGTCAAYGGIPAAKGNLTGATGVRDFFKENDINTPVVNIPGCPPH
D. autotrophicum HRM2     EMTTELGKMAGSVLAVGTCAAYGGIPAAKGNLTQATGVQNVFKKFDITTPVVNIPGCPPH
D. postgatei 2ac9         DLVKEVAPMAGSCLAVGTCAAYGGIPAAKGNVTGATGCRDFFAANSINTPIVNIPGCPPH
108                       DMVKELAPMAGSVLAVGTCAAYGGIPAAKGNVTGATGVRDFFKANDINTPVVNIPGCPPH
D. phosphitoxidans        DMVQELAPMAGSVLAVGTCAAFGGIPAAKGNVTGATGVRDFFKSKDITTPVVNIPGCPPH
139                       EVIKELATKAAAVLAVGTCAAYGGIPAAEGNLTGAMG-RDFFEENG-KTPVVNIPGCPPH
```

**Fig. S9**

```
                                250       260       270       280       290       300
                        ....|....|....|....|....|....|....|....|....|....|....|....|
D. vulgaris Hilden.     PDWIVGTVVLA--------LDAIKKNGL-------EGGLAE----VVKVLDSDGRPTPFF
102                     PDWIVGTVVVA--------LNAIKENGL-------EAGLAE----VVKLLDADGRPTP-Y
D. vulgaris Miyazaki F  PDWIVGTVVVA--------LNAIKAKGL-------AAGLGD----VVKLLDADGRPTPFY
115                     PDWIVGTVVVA--------LDAIKENGL-------EAGLAE----VVKLLDADGRPTP-Y
D. alaskensis G20       PDWIVGTVVVA--------LDAIKKHGL-------QGGLGE----VVKLLDGDGRPTPFY
123                     PDWIVGTIVVA--------LNAIKENGL-------EAGLAE----VVKLLDADGRPMPY-
D. piger                PDWIVGTIALA--------LQKIKEKGL-------EAGLAE----VVSLLDSEGRPLPFY
131                     PDWIVGTIVVA--------LNAIKENGL-------EAGLAE----VVKLLDADGRPMPY-
B. wadsworthia 3-1-6    PDWIVGTIGLG--------LQALATNTL---------GLL-----VKQGLDANGRPKAFY
136                     PDWIVGTLVVA--------LNAIKENGL-------EDGLAE----VVKILDDDGRPMLF-
133                     PDWMVGTLVVA--------LNAIKENGL-------EDGLAE----VVKILDDDGRPMLF-
132                     PDWMVGTLVVA--------LNAI----L-------EHGLAE----VVKILDDDGRPMLF-
D. baculatum            PDWMVGTLVAA----------------WSHVLNPTEHPLPE--------LDDDGRPLLFF
D. africanus WalvisBay  PDWIIGSLAFA----------------L-------EHGVEATAKI----LDAEGRPSVFF
96                      PDWMVGTLVVAINAIEEKG--------L-------QGGLAEVVKI----LDDDGRPTP-F
D. hydrothermalis       PDWMVGTLVVAINAIEEKG--------L-------QGGLAEIVTI----LDDEGRPTPFF
D. salexigens           PDWMVGTLVVAINAIEAKG--------L-------QGGLAEVVKI----LDDNGRPTPFF
121                     PDWIVGTIVHL----------------L-------KEGLPE--------LDDDGRPMLFF
D. autotrophicum HRM2   PDWIVGTIAHL----------------L-------TKGLPE--------LDENGRPLLFF
D. postgatei 2ac9       PDWIVLSIVHL----------------L-------EKGIPE--------LDDEGRPMLFF
108                     PDWIVGSIVHL----------------L-------KEGIPE--------LDDDGRPMLFF
D. phosphitoxidans      PDWIVGSIVHL----------------L-------NAGIPE--------LDYDGRPTLFF
139                     PDWIVGTLV----------------HVL-------EDGLPE--------LDDDGRPMLF-

                                310       320       330       340       350       360
                        ....|....|....|....|....|....|....|....|....|....|....|....|
D. vulgaris Hilden.     GRNIHENCPYLDKYDEGVMSATFTDKVGCRYDLGCKGPMTMADCFERKWNGGVNWCVQNA
102                     GRNIHENCPYLDKYDEGKMSETFT-KDGCRYD-GCKGPMTMSDCFERKWNGGVNWCVHNA
D. vulgaris Miyazaki F  GRNVHENCPYLEAYDAGKMCETFTKKEGCRYDLGCKGPMSMCDSFERKWNGGVNWCISNA
115                     GRNIHENCPYLDKFDEGKMSETFT-KDGCRYD-GCKGPTTMSDCFERKWNSGVNWCVDNA
D. alaskensis G20       GTNIHDNCPYLPQFEEYVMSETFTQKDGCRYELGCKGPSTMADCYKRKWNNGVNWCISNA
123                     GRNIHENCPYLDKFDEGKLSETFT-KDGCRYD-GCKGPATNSDCFERKWNSGVNWCVDNA
D. piger                GRNVHENCPYLGKYDEGKFSATFTEKDGCRYDLGCKGPGAYCDSFERKWN-GVNWCVANA
131                     GQNIHENCPYLDKFDEGKLAETFT-KDGCRYD-GCKGPATNSDCFERKWNSGVNWCVDNA
B. wadsworthia 3-1-6    -KNVHMNCPHLSAFEAGHMVKTMSDKDGCRFSMGCKGPRSACDSFERKWNNGVNWCVNNA
136                     GENIHDNCPYLDKFDNDKFAETFT-KDGCRYD-GCKGPATNSDCFKRKWNSGVNWCVDNA
133                     GENIHDNCPYLDKFDNDKFAETFT-KDGCRYD-GCKGPATNSDCFKRKWNSGVNWCVENA
132                     GENIHDNCPYLDKFDNDKFAETFT-KAGCKYD-GCKGPATNSDCFKRKWNNGVNWCVENA
D. baculatum            GDNIHENCPYLDKYDNSEFAETFTKP-GCKAELGCKGPSTYADCAKRRWNNGINWCVENA
D. africanus WalvisBay  GENIHDNCPYLEHFENDNFAATFTQAG-CKYNLGCKGPACNSDCFKRKWNSGMNWCVENA
96                      GENIHDNCPYLEAFDNDEYAEIFT-PDKCRYE-GCKGPSANSDCFKRKWNGGVNWCVENS
D. hydrothermalis       GENIHDNCPYLEAFDNDEYAEVFTDPEKCRYELGCKGPSANSDCFKRKWNGGVNWCVENS
D. salexigens           GENIHDNCPYLEAFDNDEYAEIFTDPVKCRYELGCKGPSANSDCFKRKWNGGVNWCVENS
121                     GENIHDNCPYLDDFDEDIFAETFTDNKGCRMDLGCKGPDTYADCFKRKWNSGLNWCVDNA
D. autotrophicum HRM2   GENIHENCPYLDYFDQDIYSKTFTDKKGCRMELGCKGPDTYADCFRRKWNSGLNWCIENA
D. postgatei 2ac9       GENIHDNCPRLKMYEADQLSQTLSDPKGCRINLGCKGPSTYADCYKRKWNSGLNWCVDNA
108                     GENIHDNCPYLDDYDADLLAETLSDPKGCRMDLGCKGPDTYADCYKRKWNSGLNWCVDNA
D. phosphitoxidans      GENIHDNCPHLDDYDAAFMAATLSDPKGCRMDLGCKGPDTYADCYQRKWNSGLNWCVNNA
139                     GENIHDNCPYLDKFDNDKFAETFT-KDGCRFD-GCKGPATYADCFKRKWNSGVNWCVDNA
```

**Fig. S9**

```
                                  370       380       390       400       410       420
                              ....|....|....|....|....|....|....|....|....|....|....|....|
D. vulgaris Hilden.           VCIGCVEPDFPDGKSPFYQA--MSGCTPK--AAPA-GAT-------GRTTIAIDPVTRIE
102                           VCIGCVEPDFPD-KSPFYQ---MSGCTPK--AAPA-GAT-------GKTTIAIDPVTRIE
D. vulgaris Miyazaki F        VCIGCVEPDFPDGKSPFYSA--MSGCTPK--AAPA-GAT-------GKATIAIDPVSRIE
115                           VCIGCVEPDFPD-KSPFYE---MSGCTPK--TAPA-GAT-------GKTTIAIDPVTRIE
D. alaskensis G20             VCIGCVEPDFPDGKSPFYES--MSGCTNK--MAAG-GVS-------GKTKIAIDPVTRIE
123                           VCIGCVEPDFPD-KSPFYEM--T---TPK--TAPA-GAN-------GKTTIAIDPVTRIE
D. piger                      ICIGCTEPSFPDGQSPFYSN--MA----------------------KATIAIDPVTRIE
131                           VCIGCVEPDFPD-KSPFYEM--T---TSK--TAPA-GAN-------GKTTIAIDPVTRIE
B. wadsworthia 3-1-6          TCIGCTSPTFPDGQSPFYVN--MS----------------------KTVIAIDPVTRLE
136                           VCIGCVEPGFPD-MSPFYEM--T---ASK--TAPA-GAD-------GKIKIAIDPVTRIE
133                           VCIGCVEPGFPD-MSPFYEM--M---ASK--TAPA-GAD-------GKIKIAIDPVTRIE
132                           VCIGCVEPGFPD-MSPFYEM--M---ASK--TAPA-GAD-------GKVKISIDPVTRIE
D. baculatum                  VCIGCVEPDFPDGKSPFYVAE-VS----Q--AATP-AAD-------GKVKISIDPLTRVE
D. africanus WalvisBay        VCTGCAEPGWPDNFSPFYESM-MS--EKN-------------------VKISIDPVTRIE
96                            VCIGCVEPGFPD-MSPFYE-G-MS--SKS--HAPA-GKD-------GKIKIAIDPVTRIE
D. hydrothermalis             VCLGCVEPGFPDEMSPFYEAG-MS--SKS--HAPA-SKD-------GKIKIAIDPVTRIE
D. salexigens                 VCIGCVEPGFPDEMSPFYEAG-MS--SKS--HAPA-GKD-------GKIKIAIDPVTRIE
121                           VCIGCVEPGFPDAMSPFYEPA-MAGCKPE--AAPAGATG-------KKIKIAIDPVTRIE
D. autotrophicum HRM2         VCIGCVEPGFPDASSPFYEQS-MAGCKPE--AVPVGAAG-------KKIKVAIDPVTRIE
D. postgatei 2ac9            VCIACVEPGFPDQSSPFYEPA-MSGSKP--------TTGSVGVDSTKKLKISIDPITRIE
108                           VCIGCVEPGFPDAMSPFYEPA-MAGCKPQ--AEPA-ATG-------KKIKIGIDPVTRIE
D. phosphitoxidans            VCIGCVEAGFPDAMSPFYEPAYMAGCQPQ--AEPA-QTG-------KKIKIGIDPVTRIE
139                           VCIGCVEPGFPD-MSPFYEM--T---ACKPATAPA-GAD-------GKIKIAIDPVTRIE

                                  430       440       450       460       470       480
                              ....|....|....|....|....|....|....|....|....|....|....|....|
D. vulgaris Hilden.           GHLKAEVVVENGKVVDARLSGGMYRGFETILRGRDPRDASQIVQRICGVCPTAHSTASVL
102                           GHLKAEVVVENG-VVDARLSGGMYRGFETILRGRDPR-ASQIVQRICGVCPTAHSTASVL
D. vulgaris Miyazaki F        GHLKAEVTVENGVVVDARLSGGMYRGFETILRGRDPRDASQIVQRICGVCPTAHSTASCM
115                           GHLKAEVVVENG-VVDARLSGGMFRGFENILRGRDPR-ASQIVQRICGVCPTAHSTASVL
D. alaskensis G20             GHLKAEVVVEGGKVVDAHISGGMFRGFENILRGRDPRDASQIVQRICGVCPTAHSTASVM
123                           GHLKAEVVVEDG-VVDARLSGGMFRGFENILSGRDPR-ASQIVQRICGVCPTAHATASAL
D. piger                      GHLKAQVVVENGKVVDAHLTGGMFRGFEQILKGRDPRDSTQIVQRICGVCPTSHAMASAL
131                           GHLKAEVEVEDG-VVDARLSGGMFRGFENILSGRDPR-ASQIVQRICGVCPTAHATASAL
B. wadsworthia 3-1-6          GHLKVEVQVEDGKVADAWITGGMFRGFEAILRGRNPRDASQIVQRICGVCPVAHATASSL
136                           GHLKAEVEVEDG-VVDAWFSGGMFRGFENILIGRDPR-ASQIVQRICGVCPTAHATASAL
133                           GHLKAEVEVEDG-VVDAWFSGGMFRGFENILIGRDPR-ASQIVQRICGVCPTAHSTASAL
132                           GHLKAEVEVEDG-VVDAWFSGGMFRGFENILIGRDPR-ASQIVQRICGVCPTAHSTASAL
D. baculatum                  GHLKIEVEVKDGKVVDAKCSGGMFRGFEQILRGRDPRDSSQIVQRICGVCPTAHCTASVM
D. africanus WalvisBay        GHLKAEVKVENGVVTDAWMSGGMFRGFENILIGRDPRDATQIVQRICGVCPTAHSTASCL
96                            GHLKAEVVVKDG-VVDAWFSGGMYRGFENILIGRDPR-AAQLTQRLCGVCRTAHSTASTL
D. hydrothermalis             GHLKAEVVVKDGKVVDAWLSGGMYRGFENILVGRDPRDAAQLTQRLCGVCPTAHSTASCL
D. salexigens                 GHLKAEVVVKDGKVTDAWLSGGMYRGFENILIGRDPRDAAQLTQRLCGVCPTAHSTASTR
121                           GHLKAEVEVEDGKVVDARFSGGMFRGFENILNGRDPRDATQIVQRICGVCPTAHATASAL
D. autotrophicum HRM2         GHLKVEVEVKGGKVVDARCFGGMFRGFENILTGRDPRDATQIVQRICGVCPTAHATASSL
D. postgatei 2ac9            GHLKAEVEVKNGVVVDARMSGGMYRGFEQILVGRDPRDAVQITQRICGVCPTAHATASSL
108                           GHLKAEVEVEDGKVVDARISGGMYRGFEQILNGSDPRDATQITQRICGVCPTAHATASAL
D. phosphitoxidans            GHLKAEVEVAGGKVVDAHITGGMYRGFEQILYGRDPRDATQITQRICGVCPTAHATASAL
139                           GHLKAEVEVEDG-VVDAWFSGGMFRGFENILIGRDPR-AAQIVQRICGVCPTAHATASAL
```

**supplementary Fig. S9**

```
                                490       500       510       520       530       540
                        ....|....|....|....|....|....|....|....|....|....|....|....|
D. vulgaris Hilden.     ALDEAFGAKVPNNGRITRNLIFGANYLQSHILHFYHLSAQDFVQGPDTAPFVPRFPKSDL
102                     ALDD-FGVKVPTNGRI-RNLIFGANYLQSHILHFYHLAAL-FVQGPDTAPFV-RFAKPDL
D. vulgaris Miyazaki F  ALDNAFKVKVPTNGRLTRNLTFGANYLQSHILHFYHLAALDFVQGPDSAPFVPRFAKPDL
115                     ALDD-FGVKVTTNGRI-RNLILGANYLQSHILHFYHLAAL-FVQGPDTAPFV-RFAKPDL
D. alaskensis G20       ALDKAFGVKVTTNGRLTRNLILGANYMQSHILHFYHLAALDFVQGPETAPFVPRFKNPDL
123                     ALDD-FGVKVTTNGRI-RNLILGANYLQSHILHFYHLAAL-FVQGPDTAPFV-RFAKPDL
D. piger                AQEDAFNIKVTGNGRITRNLILGANYLQSHILHFYHLAALDFVAGPDTAPFVPRFAQPDL
131                     ALDD-FGVKVTTNGRI-RNLILGANYLQSHILHFYHLAAL-YVQGPDTAPFV-RYDNPDL
B. wadsworthia 3-1-6    AIEAVCGVEVPENGRIARNLMLAGNYLQSNILHFYHLGGQDYFHGPDTVPFIPRYRNPDL
136                     ALDD-FGVKVTTNGRI-RNLILGANYLQSHILHFYHLAAL-YVNGPDMAPFV-RYDNPDL
133                     ALDD-FGVKVTTNGRI-RNLIFGANYLQSHILHFYHLAAL-YVNGPDIAPFV-RYDNPDL
132                     ALDD-FGVKVTTNGRI-RNLIFGANYLQSHILHFYHLAAL-YVNGPDIAPFV-RYDNPDL
D. baculatum            AQDDAFGVKVTTNGRITRNLIFGANYLQSHILHFYHLAALDYVKGPDVSPFVPRYANADL
D. africanus WalvisBay  ALDDAFGVKLTTNGRVTRNLIFGANYLQSHILHFYHLAALDYVAGPDVAPFVPRYEKSDL
96                      ALDD-FGVKLTTNGRV-RNLIFGANYLQSHILHFYHLAAL-FVSGPGKAPFV-RFEHPDL
D. hydrothermalis       ALDDAFGAKITTNGRVTRNLIFGANYLQSHILHFYHLTALDFVRGPGKAPFVPRFEQPDL
D. salexigens           ALDDAFGVKLTTNGRVTKNLIFGANYLQSHILHFYHLAALDFVRGPGKAPFVPRFEQPDL
121                     ALDDAFGVKLTNNGRIARNLILGANFLQSHILHFYHLAALDYVNGPDTAPFIPRYDNNDM
D. autotrophicum HRM2   ALDDAFGVKLTNNGRVARNLILGANFLQSHILHFYHLAALDYVNGPEVAPFIPRYKNNDV
D. postgatei 2ac9       ALDDAFGVTLTDNGRIARNLILGANFIQSHILHFYHLAALDYVNGPDTAPFIPRYKHNDI
108                     ALDDAFGVKLTDNGRIARNLILGANFLQSHILHFYHLAALDYVNGPDTAPFIPRYDNNDM
D. phosphitoxidans      ALDDAFGVELTDNGRIARNLILGANFLQSHILHFYHLAALDYCNGPDTAPFIPRYANNDM
139                     ALDD-FGVKVTNNGRI-RNLILGANYLQSHILHFYHLAAL-YVNGPDMAPFV-RYDNPDL

                                550       560       570       580       590       600
                        ....|....|....|....|....|....|....|....|....|....|....|....|
D. vulgaris Hilden.     RLSKELNKAG---------VDQYIEALEVRRICHEMVALFGGRMPHVQGQVVGGATEIPT
102                     RLPKEMNKA----------VDQYLEALEVRRICHEMVAL-GGRMPHVQGQVVGGATEIPT
D. vulgaris Miyazaki F  RLPKDINAAA---------VDQYLEALEVRRICHEMVAMFGGRMPHVQGQVVGGTTEIPT
115                     RLPKEMNKV----------VDQYLEALEVRRICHEMVAL-GGRMPHVQGQVVGGATEIPT
D. alaskensis G20       RLPSAVNQVA---------VDQYLEALEVRRICHEMVAIFGGRMPHVQGQVVGGTTEIPT
123                     RLPKEMNKV----------VDQYLEALEVRRICHEMVAL-GGRMPHVQGMVVGGATEIPT
D. piger                RLPPEANKVG---------VDQYLEALEVRRIAHEMVALFGGRMPHVQGIVPGGATEMPT
131                     RLPKEMNKV----------VDQYLEALEVRRICHEMVAL-GGRMPHVQGMVVGGATEIPT
B. wadsworthia 3-1-6    RLSEEQNTLA---------MDEYIEALEVRQVCHQLVALFGGRMPHLQGILGGGAAQIPD
136                     RLAKEINKV----------VDQYLEALEIRRICHEMVAL-GGKMPHVQGMVVGGATEIPT
133                     RLAHEINKV----------VDQYLEALEIRRICHEMVAL-GGKMPHVQGMVVGGATEIPT
132                     RLAHEINKV----------VDQYLEALEIRRICHEMVAL-GGKMPHVQGMVVGGATEIPT
D. baculatum            -LTDRIKDGAKADATNTYGLNQYLKALEIRRICHEMVAMFGGRMPHVQGMVVGGATEIPT
D. africanus WalvisBay  RLTPELNKVA---------VDQYLEALNVRLVAHEMVALFGGKMPHVSGQVVGGTTEIPS
96                      RLDEKTNKV----------VDQYVKALEIRRICHEMVAL-GGKMPHVSGQVVGGATEIPT
D. hydrothermalis       RLDEKTNAVA---------VDQYIKALEIRRICHEMVALFGGKMPHISGQVVGGTTEIPT
D. salexigens           RLDEKTNKVA---------VDQYVKALEIRRICHEMVALFGGKMPHVSGQVVGGATEIPT
121                     RLPKEINEVA---------VDQYLEALEIRKICHEMVALFGGKMPHVQGIVVGGTTEIPT
D. autotrophicum HRM2   RLDKATNQVG---------VDQYLEALEIRKICHEMVALLGGKMPHVQGIVVGGTTEIPT
D. postgatei 2ac9       RVSKDINDLC---------VGQYLEALEIRKICHEMVALLGGKMPHVQGIVVGGTTEIPT
108                     RLPKEINDVA---------VGQYLEALEIRKICHEMVALLGGKMPHVQGIVVGGTTEIPT
D. phosphitoxidans      RLPKELNDVA---------VGQYLEALEIRKICHEMVALLGGKMPHVQGIVVGGSTEIPT
139                     RLAKEINKV----------VDQYLEALEIRRICHEMVAL-GGKMPHVQGMVVGGATEIPT
```

**supplementary Fig. S9**

```
                                    610       620       630       640       650       660
                               ....|....|....|....|....|....|....|....|....|....|....|....|
D. vulgaris Hilden.            KEKLVEYAARFKKVRDFVEQKYVPVVYTIGSKYKDMFKVGQGFKAALCVGAFPLDNSGKK
102                            KEA-VEYAARFKKVREFVEEKYVPV-YTVGTVYKDLFKFGQGYKNCISFGAFPLNDDGNE
D. vulgaris Miyazaki F         KEALVEYAARFKKVREFVEKKYVPVAYTVGAAYKDLFKFGQGYKNCISFGAFPLNDAMTE
115                            KEA-VEYAARFKKVRKFVEEKYVPV-YIVGTVYKDLFKFGQGYKNCISFGVFPLNDDGKE
D. alaskensis G20              KEALVEYAARFKKVRKFVEEKYVPVVYLVGSVYKDLFAFGQGYKNCVAFGVFPLNDEGTE
123                            KEA-VEYAARFKKVRKFVEEKYVPV-YIVGSVYKDLFKIGQGYKNCISFGVFPLNDDGKE
D. piger                       KEALLEYAARFKKVRQFIVEKYLPITYIVGSVYKDLFEQGQGVHGCSCFGVFPMTDDGKT
131                            KET-VEYAARFKKVRKFVEEKYVPV-YIMGSVYKDLFKIGQGYKNCISFGVFPLDDDGKE
B. wadsworthia 3-1-6           RETILEYAARMKQVRKFVENRYLPLVYTIASRYMDMFEMAHGYKNALCVGVFPLAKKGE-
136                            KEK-AEYAARFKKVRKFIEEKYVPV-YIMGSVYTDLFKIGEGYKNFIAFGVFPLDDDGKE
133                            KEK-AEYAARFKKVHKFIEEKYVPV-YIMGSVYTDLFKIGDGYKNVIAFGVFPMDDDGKE
132                            KEK-AEYAARFKKVHKFIEEKYVPV-YIMGSVYTDLFKIGDGYKNVIAFGVFPMDDDGKE
D. baculatum                   ADKVAEYAARFKEVQKFVIEEYLPLIYTLGSVYTDLFETGIGWKNVIAFGVFPEDDDYKT
D. africanus WalvisBay         QQKLDEYTKRFKLVRKFIEETYVPTVYVIGKAYADLLKVGDGYKNVISFGVFPMDDSGKE
96                             KEK-AEYASRFKEVQKFIEETYVP-VYLIGSVYKDLFKIGGGYKNAMAYGVFPMDDAGYE
D. hydrothermalis              KEKLAEYASRFKDVQKFIAEVYVPTVYLIGSVYKDLFKIGGGYKNCMAYGVFPMSDGGDD
D. salexigens                  KEKLAEYASRFKQVQKFIEETYVPTVYLIGSVYKDLFKIGGGYKNAMAYGVFPMDDAESE
121                            RENLDAYAERFKKVRKFIEEKYVPIVYLLAGPYGDLLKTGVGYKNFIAFGVFPLDDEGN-
D. autotrophicum HRM2          REKLDAYKERFKTVRKFIEERYLPLIYLLAGPYGDLLKTGTGYKNCIAFGVFPLDDAGN-
D. postgatei 2ac9              REALNAYAERFKKIKKFVMEKYIPVIYTLAGPYGDLLKTGVGHKNLVSWGVFPMDSKGN-
108                            REALNAYAERFKKVRKFIVEKYVPIVYLLAGPYGDLLKTGVGHKNLVSWGVFPLDNKGN-
D. phosphitoxidans             REALNAYAERFKKVRQFILEKYVPIVYLLAGPYGDLLKTGVGHKNLVSWGVFPLDNKGN-
139                            KEK-AEYAARFKKVSKFIEEKYVPV-YIMASVYTDLFKIGEGYKNFIAFGVFPLDDDGNE

                                   670       680       690       700       710       720
                               ....|....|....|....|....|....|....|....|....|....|....|....|
D. vulgaris Hilden.            HLFMPGVYAKGKDMPFDPSKIKEYVKYSWFAEETTG-LNYKEGKTIPAPDKAGAYSFVKA
102                            LLLKPGVYIDGKDKPFDPKQIKEYVKYSW-DDATTG-LH-KEGKTIPAPDKA-AYSFVKA
D. vulgaris Miyazaki F         LHLKRGVYIDGKDQPFDPKLIKEYVKYSWFDDATTG-LHFSQGKTVPAPNKAGAYSFVKA
115                            LLLKPGVYIDGKDKPFDPKQIKEYVKYSW-DDATTG-LH-NEGKTIPDPDKA-AYSFVKA
D. alaskensis G20              FALKPGVYMDGEDKPFDARLIKEYVKYSWFDDSTTG-LHYTEGQTIPDPEKPGAYSFVKA
123                            LLLKPGVYIDGKDEPFDPKQIKEYVKYSW-DDATTG-LH-NEGKTIPDPDKA-AYSFVKA
D. piger                       HLLKAGVFLNGRDVEFDPKKITEDLKYAWYDDATTG-KGADGAETNPNLDKKDAYSFVKA
131                            LLLKPGVYIDGKDEPFDPKQIKEYVKYSW-DDATTG-LH-NEGKTIPDPDKA-AYSFVKA
B. wadsworthia 3-1-6           QFFNAGAYINGRDEPFDGNRILEDVRYSWFEPAPSG-TPLQKSESNPQVDKEGAYSFIKA
136                            FLLKPGVYIDGKDEAFDPKQIKEYVKYSW-DDATTG-LH-SEGKTIPDPDKA-AYSFIKA
133                            FLLKPGVYIDGKDEAFDPKQIKEYVKYSW-DDASTG-LH-SEGKTIPDPDKA-AYSFIKA
132                            FLLKPGVYIDGKDEAFDPKQIKEYVKYSW-DDASTG-LH-SEGKTIPDPDKP-AYSFIKA
D. baculatum                   FLLKPGVYIDGKDEEFDSKLVKEYVGHSFFDHSAPGGLHYSVGETNPNPDKPGAYSFVKA
D. africanus WalvisBay         TLLKPGVYINGKDVAFDPAKIKEYAKYSWFEDSCSN-LHPSQGKTLPKLGKPGAYSFIKA
96                             FLLKPGVYIDGKDDGFDPKLIKEYTKYSW-TDECSD-LH-SEGKTIPDVHKK-AYSFCKA
D. hydrothermalis              FLIKSGVYVDGKDEKFDPKLIKEFTKYSWYTDECSD-LHPSEGKTIPDVHKKDAYSFCKA
D. salexigens                  FLLKPGVYIDGKDAGFDQKLIKEYTKYAWYTDECSD-LHPSKGKTIPDVHKKDAYSFCKA
121                            TLLKPGVYTDGKDKAFDPDKIKEYVKYSWFDDTTTG-LHPSEGKTVPDPDKAGAYSFIKA
D. autotrophicum HRM2          TLLKSGVFTDGKYTDFNSDNIKEYVKHSFFADNTTG-LHPSKGKTVPDPEKATGYSFIKS
D. postgatei 2ac9              TLLKPGVYTDGKDYKVDPTQIKEYVKYSWFEDSTTG-LNPTRGRTRPEPGKAGAYSFIKA
108                            TLLKPGVYTDGKDYAVDPAKIKEYVKYSWFEDTTTG-LNPTEGKTLPDPDKAGAYSFIKA
D. phosphitoxidans             TLLKPGVYTDGKDYAVDPAMIKEYVKYSWFEDSTTG-LNPTEGKTLPNPNKPGAYSFIKA
139                            -LLKPGVYIDGKDEAFDPNQIKEYVKYSW-DDDTTG-LH-SEGKTIPDPDKA-AYSFIKA
```

```
                                   730       740       750       760       770       780
                          ....|....|....|....|....|....|....|....|....|....|....|....|
D. vulgaris Hilden.       PRYDGLSLEVGPLARMWVNNPELSPVGKKLLKDLFGISAKKFRDLG-EEAAFSLMGRHVA
102                       PRYNGKPVEVGPLA-MWVT-PELSPVGQKLLKDLFGIDAKSFRD-G-DEAAFSLMGRHVA
D. vulgaris Miyazaki F    PRYNGKAVESGPVARMWITNPELSPVGQKLLKDLFKLNAKRFRDLG-DEAAFSVMGRHVA
115                       PRYNGKPVEVGPLA-MWVT-PELSPMGQKLLKDLFGIDAKSFRD-G-DEAAFSLMGRHVA
D. alaskensis G20         PRYNGKPCEVGPLARMWVANPELSPMGQKMLKEHYGIDAKRFRDIG-AEAAFSLMGRHVA
123                       PRYNGKPVEVGPLA-MWVT-PELSPMGQKLLKDLFGIDAKSFRD-G-EDAAFSIMGRHVA
D. piger                  PRYDGEVIEVGPAARMWVANAPLSEVGVKMLKEKFGIEARTIRDLG-WDKVFSIMGRHVA
131                       PRYNGKPVEVGPLA-MWVT-PELSPMGQKLLKDLFGIDAKSFRD-G-EDAAFSIMGRHVA
B. wadsworthia 3-1-6      PLYAGHRVEVGPLARMWINDKPLSPIGQRFFADMFGVRAETFRQIG-EDPAFSIMGRNVA
136                       PRYNGKPVEVGPLA-MWVT-PELSPMGQKLLKDLFGIDAKSFRD-G-EDAAFSIMGRHVA
133                       PRYNGKPVEVGPLA-MWVP-PELSPMGQKLLKDLFGIDAKSFRD-G-EDMAFSIMGRHVA
132                       PRYNGKPVEVGPLA-MWVP-PELSPMGQKLLKDLFGIDAKSFRD-G--DMAFSIMGRHVA
D. baculatum              PRYKDKPCEVGPLARMWVQNPELSPVGQKLLKELYGIEAKNFRDLG--DKAFSIMGRHVA
D. africanus WalvisBay    PRYDGHPVEVGPLARMWVSNPELSPMGKKQLKDLFGINANRFRDLG--DLAFSIMGRHVA
96                        SRYNGKAVEVGPLA-MWVH-PELSPMGKKQLKDLFGIEAKIFRD-GE-DMAFSLMGRHVA
D. hydrothermalis         SRYNGNAVEVGPLARMWIHNPELSPMGKKQLKDLYGITAKNFRDLGE-DMAFSLMGRHVA
D. salexigens             SRYNGKAVEVGPLARMWVHNPELSPMGKKQLKDLFGIEAKMFRDLGE-DMAFSLMGRHVA
121                       PRYNGKPHEVGPLARMWVTNPELSATGQKAL------GVKRLSDIG--DAAFSILGRHVA
D. autotrophicum HRM2     PRYNGKPHEVGPLARMWITNPELSATGQKAL------GVKRMRDIG--DAAFSILGRHIA
D. postgatei 2ac9         PRYNGKPHEGGPLARMWATNPELSKTGQEAL------GVKKLRDIG--DACFSILGRHVA
108                       PRYNGKPHEGGPLARMWATNPELSATGQKAL------GVKRLSDIG--DACFSILGRHVA
D. phosphitoxidans        PRYNGKPHEGGPLARMWATNPELSATGQKEL------GVTRLRDIG--DACFSILGRHVA
139                       PRYNGKPLEVGPLA-MWVT-PELSPMGQKLL------DAKSFRD-G--DAAFSIMGRHVA

                                   790       800       810       820       830       840
                          ....|....|....|....|....|....|....|....|....|....|....|....|
D. vulgaris Hilden.       RAEETYYMLGAIE--GWLKEIKAGEDTVVMPAVPASAEGTGFTEAPRGSLLHYVKVKDSK
102                       RAEETYYMLNAIE--RWLKEVKAGEET-VASEIP-SAEGIGFTEAPRGSLLHYINIK-SK
D. vulgaris Miyazaki F    RAEETYYMLSAIE--RWLKEVKAGEETFAAAEIPASSEGVGFTEAPRGSLVHYINIKDQK
115                       RAEETYLIPNAIE--RWLKEVKPGEET-VPSEIP-SAEGIGFTEAPRGSLLHYINIK-YK
D. alaskensis G20         RAEETWLTTNYIE--RWLKEVVPGAETYVPSEIPEQAEGTGFTEAPRGSLLHYIDIKDSV
123                       RAEETYLIANAIE--RWLKEVKPGEET-VPSEIP-SAEGIGFTEAPRGSLLHYINIK-YK
D. piger                  RAEEALLIANAVE--GWLKEVKPDGETFTPFEIPQSAEGYGCSEAPRGSLVHYIRVKDQK
131                       RAEETYLIANAIE--RWLKEVKPGEET-VASEIP-TAEGIGFTEAPRGSLLHYINIK-YK
B. wadsworthia 3-1-6      RVEEVYQTLGMIE--YWLHELEPGAQTFALPEVPQAGEGIGFTEAPRGALCHYMRVKNGV
136                       RAEETYLVANAIE--RWLKEVKPGEET-VASEIP-TAEGIGFTEAPRGSLLHYINIK-YK
133                       RAEETYLVANAIED-RWLKEVKPGEET-VTSEIP-TAEGIGFTEAPRGSLLHYINIK-YK
132                       RAEETYLVANAIED-RWLKEVKPGEET-VTSEIP-TAEGIGFTEAPRGSLLHYINIK-YK
D. baculatum              RAEETWLTAVAVE--KWLKQVQPGAETYVKSEIPDAAEGTGFTEAPRGALLHYLKIKDKK
D. africanus WalvisBay    RAEETYLVAKAIE-ERWLKEVKPGKETFVAASIPDSAEGIGFTEAPRGSLLHYVNIKDKK
96                        RAEEAYMVANAIE-DAWLKEVKPGEET-VKTEIP-SAEGLGLTEAPRGSLLHYINIK-SK
D. hydrothermalis         RAEESYLVANAIG-DIWLGEVKEGEETYVKTTMPESGEGVGLTEAPRGSLLHYINIKDSK
D. salexigens             RAEEAYMVANAIQ-DAWLKEVQPGEETYVKTEIPESAEGLGLTEAPRGSLLHYINIKDSK
121                       RAEETLLVAKAME--HWLDEAKPGKETFVAAIPENAEGIGLTEAPRGALLHYIDIKNYK
D. autotrophicum HRM2     RAEETLLVAMQME--RWLEEAKPGLETFVAAPIPENAEGIGLTEAPRGALLHYIDIKNSV
D. postgatei 2ac9         RAEETLLVAKAVE--QWIAQATPGKETFVPAAIPENAEGLGMTEAPRGALLHYVEIKNSV
108                       RAEETLLVAKAME--QWLTQAKPGKETFVPAAIPENAEGLGMTEAPRGALLHYIDIKNYK
D. phosphitoxidans        RAEETALVAAAME--EWLTQAQPGKETFVPAPIPDSAQGLGMTEAPRGALLHYIDIKDQK
139                       RAEETYLVANAIE--HWLKEVKPGEET-VASEIP-NAEGIGFTEAPRGALLHYINIK-YK
```

**supplementary Fig. S9**

```
                                850       860       870       880       890       900
                           ....|....|....|....|....|....|....|....|....|....|....|....|
D. vulgaris Hilden.        IDNYQIVSASLWNCNPRDDMGQRGAVEEALIGIPVDDIQNPVNVARLIRAFDPULGCAVH
102                        IDNYQIVSATLWNCNPRDDMGQRG-VEEALIGIPVPDINNPVNVARLIRAFDPULGCAVH
D. vulgaris Miyazaki F     IDNYQIVSATLWNCNPRDDSGQLGPVERALVGTPVPDISNPVNVARVIRAFDPULGCAVH
115                        IDNYQIVSATLWNCNPRDDMGQRG-VEEALIGIPVPDINNPVNVARLIRAFDPULGCAVH
D. alaskensis G20          ISNYQIVSATLWNCNPRDDMGNRGPVEEALIGTPVPDLENPVNIARLIRAFDPULGCAVH
123                        IDNYQIVSATLWNCNPRDDMGQRG-VEEALIGIPVPDINNPVNVGRIIRAFDPULGCAVH
D.piger                    IDSYQIISATLWNCSPRDDKGRRGPLEEALIGVQVPDINNPVNVGRTIRAFDPULGCAVH
131                        IDNYQIVSATLWNCNPRDDMGQRG-VEEALIGIPVPDINNPVNVGRIIRAFDPULGCAVH
B. wadsworthia 3-1-6       IDDYAVVAASMWNCSPRDDAGKRGAVEEALIGVPVPEVDSPVNVGRVIRAYDPULGCAVH
136                        IDNYQIVSATLWNANPRDDMGQRG-MEEALIGIPVPDIKNPVNVGRIIRSFDPULGCAVH
133                        IDNYQIVSATLWNANPRDDMGQRG-IEEALIGIPVPDIKNPVNVGRIIRSFDPULGCAVH
132                        IDNYQIVSATLWNANPRDDMGQRG-IEEALIGIPVPDIKNPVNVGRIIRSYDPULGCAVH
D. baculatum               IENYQIVSATLWNANPRDDMGQRGPIEEALIGVPVPDIKNPVNVGRLVRSYDPULGCAVH
D. africanus WalvisBay     IANYQIVSATLWNANPRDDMGKRGPMEQALIGTPVPDAKNPVNVGRVIRAYDPULGCAVH
96                         TANYQMIPATLWNSTPRDDKGQRG-IEEALVGTPVPDPKNPVDISRIIRSFDPULGCAVH
D. hydrothermalis          TANYQMIPATLWNSTPRDDKGMRGTIEEALVGCPVPDPSSPVDISRIIRSFDPULGCAVH
D. salexigens              TANYQMIPATLWNSTPRDDKGHRGTIEEALVGTPVPDPKNPVDISRIIRSFDPULGCAVH
121                        IANYQITSATIWNANPRDDMGQRGPIEEALIGIPVPDIDNPVNVGRLIRSFDPULGCAVH
D. autotrophicum HRM2      ISNYQITSATIWNANPRDDMEQRGPIEQALIGIPVPDIDSPVNVGRLIRSFDPULGCAVH
D. postgatei 2ac9          ISNYQITSATIWNANPRDDMGQRGPIEEALIGVPVPDVDNPVNVGRLIRAYDPUMGCAVH
108                        IANYQITSATIWNANPRDDMGQRGPIEEALIGVPVPDVDNPVNVGRLIRSFDPULGCAVH
D. phosphitoxidans         IANYQITSATIWNANPRDDMGQRGPIEEALIGVPVPDVNSPVNVGRLIRSFDPULGCAVH
139                        IDNYQIVSATIWNANPRDDMGQRG-MEEALIGIPVPDIDNPVNVGRIIRSFDPULGCAVH

                                910
                           ....|....|....|....
D. vulgaris Hilden.        VLHAESGKVAVIEVK----
102                        VLHAESGKVSVVEVK----
D. vulgaris Miyazaki F     VLHAESGKVSVVEVK----
115                        VLHAESGKVSVVEVK----
D. alaskensis G20          VLHAESGKVSVVEF-----
123                        VLHAETGKVSVVEVE----
D.piger                    VLHAETG------------
131                        VLHAETGKVSVVEVE----
B. wadsworthia 3-1-6       VLHAKAE------------
136                        VLHAETGKEHVVEVE----
133                        VLHAETGEEHVVNVE----
132                        VLHAETGEEHVVNVE----
D. baculatum               VLHAETGEEHVVNID----
D. africanus WalvisBay     VLHAETGEKTVVTVE----
96                         VLHAETGEEHVVHVGEGC-
D. hydrothermalis          VLHAETGEEHVYHVGEGC-
D. salexigens              VLHAETGEEHVVHVGEGC-
121                        VLHAETGKTIVVDVP---L
D. autotrophicum HRM2      VLHAETGKTNVVEIP---L
D. postgatei 2ac9          VLDADTGKQIKVEVP---L
108                        VLDAETGKTIKVDVP---L
D. phosphitoxidans         VLDVETGRTVKVDVP---L
139                        VLHAETGKENVVEVE----
```

**Supplementary Fig. S9: Amino acid sequence alignment and identification of residues involved in the cavity.** Amino acid residues that have more non-synonymous substitutions ($\delta N - \delta S > 0$) are marked in yellow. The residues constituting cavities are designated in red. Assemblage names in the left column are grouped as terrestrial origin (highlighted in yellow), clinical isolates (brown letters), deep-sea marine vent origin (highlighted in blue), and sediment origin (highlighted in green). Ternary structural models start with R78 and end at H900.

| MD model | z-Score | MD model | z-Score | MD model | z-Score |
|---|---|---|---|---|---|
| *D. vulgaris* Hildenborough | -11.71 | *D. baculatum* | -11.22 | *D. postgatei* 2ac9 | -9.86 |
| 2wpn.pdb (*D. vulgaris* H. ) | -11.59 | 1cc1.pdb (*D. baculatum*) | -10.64 | *D. phosphit-oxidans* | -9.99 |
| *D. vulgaris* Miyazaki F | -10.69 | *D. africanus* Walvis Bay | -11.20 | *D.autotrophicum HRM2* | -11.22 |
| *D. alaskensis* G20 | -11.38 | *D. salexigens* | -10.73 | 108 | -11.10 |
| *B. wadsworthia* 3-1-6 | -10.42 | *D. hydrothermalis* | -8.96 | 121 | -11.25 |
| *D. piger* | -11.56 | 132 | -9.69 | | |
| 102 | -10.25 | 96 | -10.30 | | |
| 115 | -10.07 | 133 | -9.92 | | |
| 123 | -9.71 | 136 | -9.75 | | |
| 131 | -9.88 | 139 | -10.42 | | |

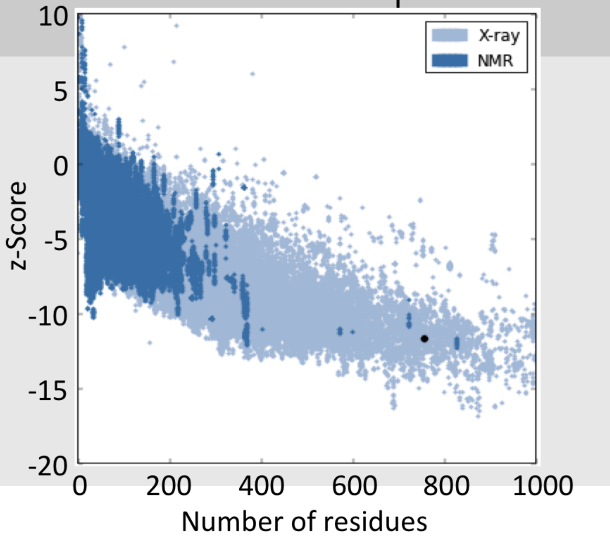


**Supplementary Table S1: Quality of MD models evaluated by ProSA**. ProSA is a knowledge-based program for the evaluation of the model accuracy. The *z*-score indicates overall model quality and measures the deviation of the total energy of the structure, which is displayed in a plot that contains the *z*-scores of all experimentally determined protein chains in current PDB. The range of z-scores between -9 and -14 is acceptable for the [NiFeSe]-Hases with 770 amino acid residue. X-ray crystallographic models 2wpn.pdb and 1cc1.pdb are also evaluated as reference.
